# Kinetics of the PASC Index in Long COVID

**DOI:** 10.1371/journal.pone.0357670

**Published:** 2026-09-08

**Authors:** Kyungsup Kwon, Choi Young Jang, Woori Kim, JuYeon Son, Euijin Chang, Sung-Han Kim

**Affiliations:** Department of Infectious Diseases, Asan Medical Center, University of Ulsan College of Medicine, Seoul, Republic of Korea; Shanghai Jiao Tong University School of Medicine, CHINA

## Abstract

**Background:**

The post‑acute sequelae of SARS‑CoV‑2 infection (PASC, “Long COVID”) remain difficult to evaluate because standardized diagnostic tools are limited. We applied the recently proposed PASC index (score ≥ 12) to describe the 12‑month trajectory of Long COVID symptoms.

**Methods:**

In this prospective cohort, adults with laboratory‑confirmed COVID‑19 were consecutively enrolled from November 2022 to February 2025. Long COVID was defined by a PASC index of ≥12 across 12 symptom domains persisting for at least 30 days post-infection. Symptom questionnaires were completed at 1, 3, 6 and 12 months after infection.

**Results:**

Among 183 participants, 48 (26.2%) met Long COVID criteria. Symptom assessments indicated that the proportion of participants meeting the Long COVID threshold declined from 27% at 1 month to 18% at 12 months, although this change was not statistically significant (p = 0.16). Participants classified as having Long COVID had consistently higher PASC index values across follow-up, while pairwise within-person comparisons showed no significant temporal changes in either the Long COVID or non-Long COVID group. These findings suggest that the overall symptom burden remained relatively stable over time, despite fluctuations in threshold status.

**Conclusion:**

During 12 months of follow-up, approximately one quarter of participants met the PASC index threshold at least once. Among participants with repeated assessments, PASC index scores showed limited within-person change, although differential non-response limits the interpretation of temporal prevalence estimates.

## Introduction

Post-acute sequelae of SARS-CoV-2 infection (PASC), referred to by various terminologies including Long COVID and Post-COVID-19 Condition (PCC), represent significant medical challenges that arise after the initial acute phase of COVID-19 infection [1]. Long COVID is not merely a transient phenomenon occurring shortly after acute infection; rather, it represents a chronic condition with sustained health impacts [2,3]. It can affect multiple organ systems, including the neurologic, gastrointestinal, and cardiovascular systems [4]. Although long COVID affects millions of people worldwide, clinicians continue to face significant challenges in accurately diagnosing and assessing these sequelae [5]. Such difficulties primarily stem from the absence of a definitive diagnostic “gold standard,” reflecting the underlying heterogeneity and complexity of pathophysiological mechanisms involved. The lack of standardized diagnostic criteria hampers effective clinical management and delays research progress in this field [6].To address this critical gap, the National Institutes of Health (NIH), through the Researching COVID to Enhance Recovery (RECOVER) initiative, funded research efforts to establish new diagnostic criterion for Long COVID, termed the PASC index [7]. The PASC index is based on self-reported symptom scoring, where individuals with scores of 12 or higher meet the diagnostic criterion for Long COVID. However, despite the availability of this novel definition, data examining the natural course and longitudinal changes based on these criterion remain scarce. Therefore, our study aims to longitudinally monitor patients diagnosed with COVID-19 using the PASC index and to evaluate how their symptom scores evolve over time.

## Methods

### Study design and participants

We conducted a prospective cohort study enrolling adults aged 18 years or older who were confirmed to have SARS-CoV-2 infection by either rapid antigen testing or polymerase chain reaction. From November 2022 through February 2025, participants underwent Long COVID evaluations, primarily through self-administered questionnaires. Although clinical visits took place in person, the survey component was completed online. Participants were recruited through advertisements both within and outside the hospital, as well as through contact with individuals visiting the hospital due to SARS-CoV-2 infection, after obtaining informed consent. Participants were monitored from enrollment through one-year post-infection. Assessments and symptom surveys were conducted at a single tertiary center in Seoul, South Korea, at 1, 3, 6, and 12months after infection. A total of 183 participants were enrolled. The distribution of participants according to the timing of their first completed questionnaire was as follows: 63 (34.4%) at 1 month, 38 (20.8%) at 3 months, 22 (12.0%) at 6 months, and 60 (32.8%) at 12 months after infection. The overall numbers of participants who completed the questionnaire at each follow-up assessment were 63 of 183 (34.4%) at 1 month, 90 of 183 (49.2%) at 3 months, 106 of 183 (57.9%) at 6 months, and 154 of 183 (84.2%) at 12 months.

### Definitions and variables

Long COVID was defined using the PASC index, based on a self-reported questionnaire evaluating 12 symptoms (S1 Table). Participants scoring 12 points or higher on this scale at least 30 days after acute infection were classified as having Long COVID, defined as having at least one occurrence of a score ≥12 at any follow-up assessment conducted from 30 days up to 1-year post-infection. Accordingly, this classification indicates that participants ever met the PASC index threshold during follow-up and does not imply that the threshold was met at every assessment. The primary outcome was defined as the proportion of patients with a PASC index of 12 or higher at each time point, and the secondary outcome was defined as the PASC index at each time point.

### Statistical analysis

Continuous variables were summarized as median with interquartile range (IQR), while categorical variables were reported as counts and percentages. Group comparisons were performed using independent t-tests, Mann-Whitney U tests, or chi-square tests, as appropriate. A two-tailed *P*-value *<* 0.05 was considered statistically significant. The primary longitudinal analysis evaluated changes in the proportion of participants with a PASC index ≥12 over time using generalized estimating equations (GEE) with an autoregressive(1) working correlation structure. Time was modeled as a categorical variable, with the 1-month assessment as the reference. Within-person changes in the continuous PASC index were evaluated using pairwise Wilcoxon signed-rank tests with Bonferroni correction for multiple comparisons. For pairwise within-person comparisons of the continuous PASC index, effect sizes were reported as matched-pairs rank-biserial correlations. Missing PASC index values were not imputed. For the longitudinal GEE analysis of the binary PASC index threshold, all available observations were included. For pairwise within-person comparisons of the continuous PASC index, analyses were restricted to participants with non-missing values at both time points being compared. To assess potential attrition bias, we compared baseline characteristics between responders and non-responders at each follow-up time point. Responders were defined as participants with a non-missing PASC index score at the corresponding assessment. We also performed a complete-case sensitivity analysis restricted to participants who completed all four follow-up assessments, applying the same paired Wilcoxon signed-rank testing framework. Separately, to address the possibility that participants below the PASC index threshold may have included subthreshold or probable Long COVID cases, we performed an additional complete-case sensitivity analysis using a more restrictive comparator definition. In this analysis, the non-Long COVID group was defined as participants who completed all four follow-up assessments and had a PASC index ≤11 at every time point. To assess health-related quality of life, EQ-5D-5L index scores were calculated using the Korean value set, and correlations between EQ-5D-5L index scores and PASC index scores were assessed using Pearson’s correlation coefficient. All statistical analyses were conducted using R version 4.4.1 (R Foundation for Statistical Computing, Vienna, Austria).

### Ethics approval

The study received ethical approval from the Institutional Review Board of Asan Medical Center (IRB No. 2022–1477). All participants provided written informed consent prior to enrollment.

## Results

### Participant characteristics and long COVID classification

Of the 183 participants enrolled during the study period, 48 (26.2%) were classified as having Long COVID (PASC index ≥12). Clinical and demographic characteristics of participants stratified according to Long COVID diagnosis based on the PASC index are summarized in Table 1. All participants experienced their initial SARS-CoV-2 infection during the Omicron-dominant era. Compared with participants without Long COVID, those diagnosed with Long COVID demonstrated significantly older age, higher proportions of male participants, increased comorbidity index scores, and a higher rate of COVID-19-related hospitalization. No significant difference in vaccination status was observed between the two groups.

**Table 1:** Demographic and clinical characteristics of participants according to PASC index.

Characteristics	Long COVID (n = 48)	Non-long COVID (n = 135)	p-value
Age (years), median (IQR)	63.5 (55.0–71.0)	41.0 (33.0–56.0)	<0.001
Male gender	30 (62.5)	40 (29.6)	<0.001
Obesity^a^	13 (27.7)	30 (22.2)	0.578
Number of infections			0.91
1	37 (78.7)	107 (79.9)	
2	10 (21.3)	25 (18.7)	
3	0	2 (1.5)	
Vaccination status^b^			0.24
Unvaccinated	5 (10.4)	8 (6.0)	
Partially vaccinated	3 (6.2)	19 (14.2)	
Fully vaccinated	40 (83.3)	107 (79.9)	
Predominant SARS-CoV-2 strain at index infection^c^			
Omicron	48 (100)	135 (100)	
Smoking	18 (38.3)	26 (19.3)	0.02
Charlson comorbidity index, median (IQR)	4.0 (2.0-5.0)	0.0 (0.0-2.0)	<0.001
Underlying disease			
COPD	5 (10.6)	1 (0.7)	0.005
Asthma	3 (6.4)	1 (0.7)	0.05
Diabetes mellitus	18 (38.3)	19 (14.1)	0.001
CKD	13 (27.7)	14 (10.4)	0.008
Chronic liver diseases	3 (6.4)	2 (1.5)	0.11
Solid tumor	10 (21.3)	14 (10.4)	0.10
Hematologic malignancy	4 (8.5)	5 (3.7)	0.24
Hematopoietic stem cell transplant	2 (4.3)	2 (1.5)	0.28
Solid organ transplant	13 (27.7)	16 (11.9)	0.02
Mental health problems	1 (2.1)	5 (3.7)	>0.999
Hospitalization for COVID-19	31 (66.0)	39 (28.9)	<0.001
Hospital treatment			
Oxygen therapy	17 (36.2)	24 (17.8)	0.02
Steroid	19 (40.4)	25 (18.5)	0.005
Remdesivir	31 (66.0)	40 (29.6)	<0.001
Tocilizumab	4 (8.5)	6 (4.4)	0.29
Baricitinib	3 (6.4)	6 (4.4)	0.709

Abbreviation: CKD, Chronic kidney disease; COPD, Chronic obstructive pulmonary disease; IQR, Interquartile range; PASC, Post-acute sequelae of SARS-CoV-2 infection

^a^ Obesity defined as body mass index > 25 kg/m².

^b^ Vaccination status defined as follows: unvaccinated (0 doses), partially vaccinated (1–2 doses), fully vaccinated (≥3 doses).^c^Predominant SARS-CoV-2 variant. Source: https://ourworldindata.org/coronavirus, accessed February 26, 2025.

### Prevalence of long COVID over time

**Fig 1:**
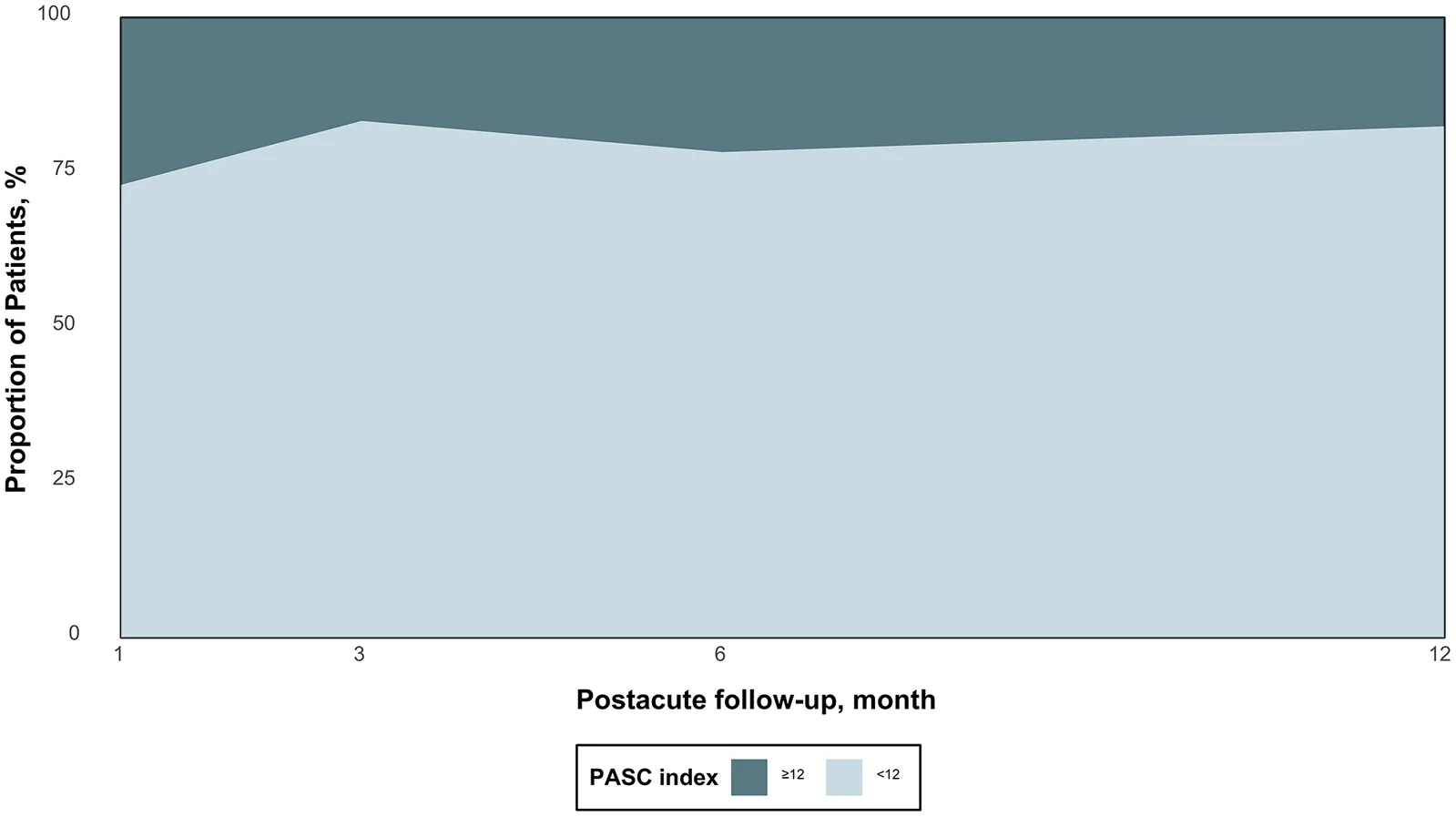
Time-dependent changes in the proportion of individuals classified as Long COVID (PASC Index ≥12).

The prevalence of the Long COVID was 27.0% (17/63) at one month, 16.7% (15/90) at three months, 21.7% (23/106) at six months, and 17.5% (27/154) at twelve months (Fig 1). In the GEE analysis using 1 month as the reference, the difference at 12 months was not statistically significant (OR = 0.64, p = 0.16). Although the odds of meeting the PASC index threshold were significantly lower at 3 months (OR = 0.46, p = 0.04), this reduction was not sustained at 6 months (OR = 0.57, p = 0.13) or 12 months.

### Temporal changes in the PASC index

**Fig 2:**
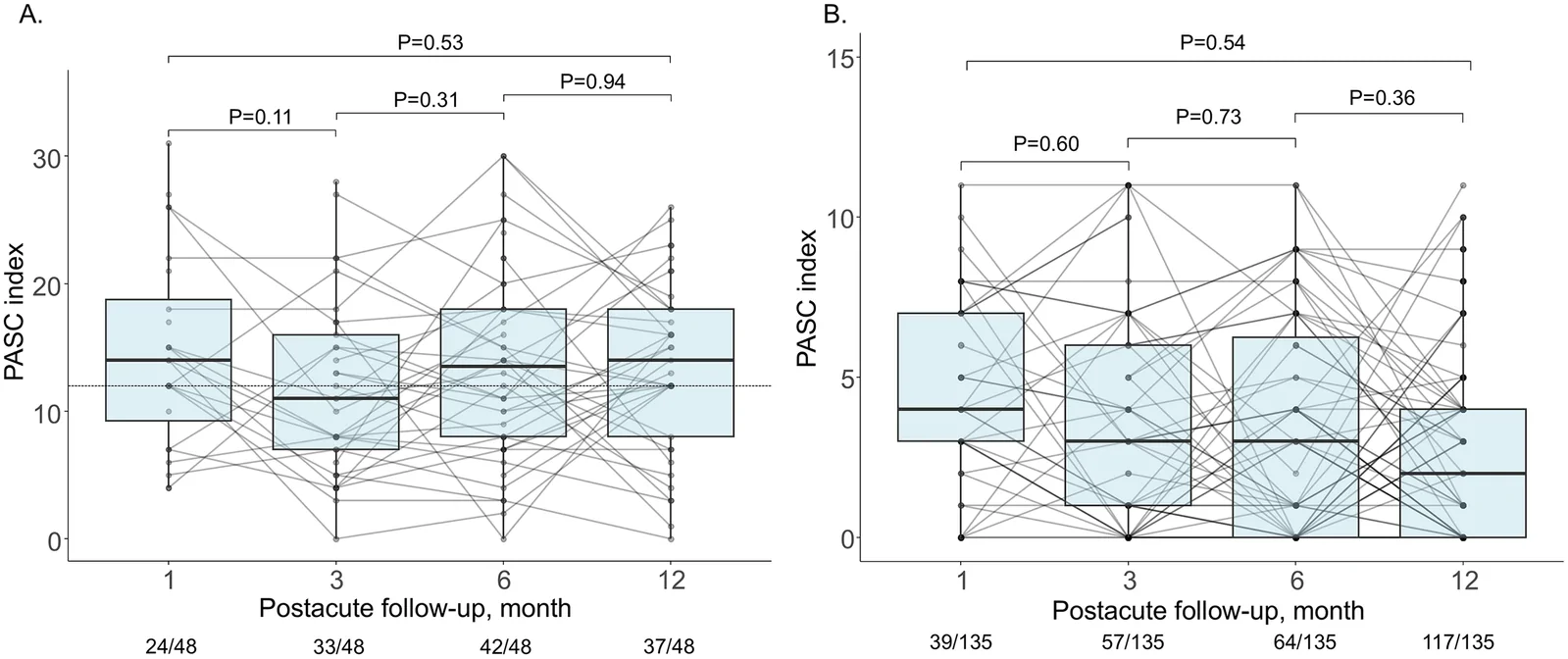
Temporal changes in the PASC index according to Long COVID status. (A) Long COVID group. (B) Non-Long COVID group. ^a^ The black dotted line indicates the PASC index threshold of 12. ^b^ Box plot: central line (median), box (IQR, interquartile range).^c^ Numbers below the X-axis indicate response counts (n).

Fig 2 shows changes in the PASC index over time according to Long COVID status. In the Long COVID group, the median PASC index was 14 (interquartile range, IQR, 9.25–18.8) at 1 month, 11 (7–16) at 3 months, 13.5 (8–18) at 6 months, and 14 (8–18) at 12 months. Pairwise within-person comparisons using paired Wilcoxon signed-rank tests showed no significant differences between any time points (1 vs. 3 months, p = 0.11; 3 vs. 6 months, p = 0.31; 6 vs. 12 months, p = 0.94; 1 vs. 12 months, p = 0.53), and none remained significant after Bonferroni correction. In the non-Long COVID group, the median PASC index was 4 (3–7) at 1 month, 3 (1–6) at 3 months, 3 (0–6.25) at 6 months, and 2 (0–4) at 12 months, and pairwise comparisons likewise showed no significant differences (1 vs. 3 months, p = 0.60; 3 vs. 6 months, p = 0.73; 6 vs. 12 months, p = 0.36; 1 vs. 12 months, p = 0.54). Effect sizes for all pairwise within-person comparisons, expressed as matched-pairs rank-biserial correlations, are presented in S3 Table. The 1-to-12-month comparisons showed small effect sizes in both the Long COVID and non-Long COVID groups, consistent with minimal overall within-person change over the full follow-up period.

### Attrition and complete-case sensitivity analyses

Attrition analyses showed differential response patterns across follow-up time points (S4–S7 Tables). Early responders tended to have greater comorbidity burden and more severe acute COVID-19-related features, whereas 12-month non-responders were older and had greater comorbidity and severity profiles than 12-month responders.

In the complete-case sensitivity analysis restricted to participants who completed all four assessments (n = 39), pairwise within-person comparisons using the Wilcoxon signed-rank test showed no significant differences in the PASC index across adjacent time points or between 1 and 12 months. The raw p-values were 0.31 for 1 vs. 3 months, 0.97 for 3 vs. 6 months, 0.78 for 6 vs. 12 months, and 0.50 for 1 vs. 12 months (S1 Fig). After Bonferroni correction for multiple comparisons, all adjusted p-values were >0.999.

### Symptom-specific frequencies and correlation with quality of life

**Fig 3:**
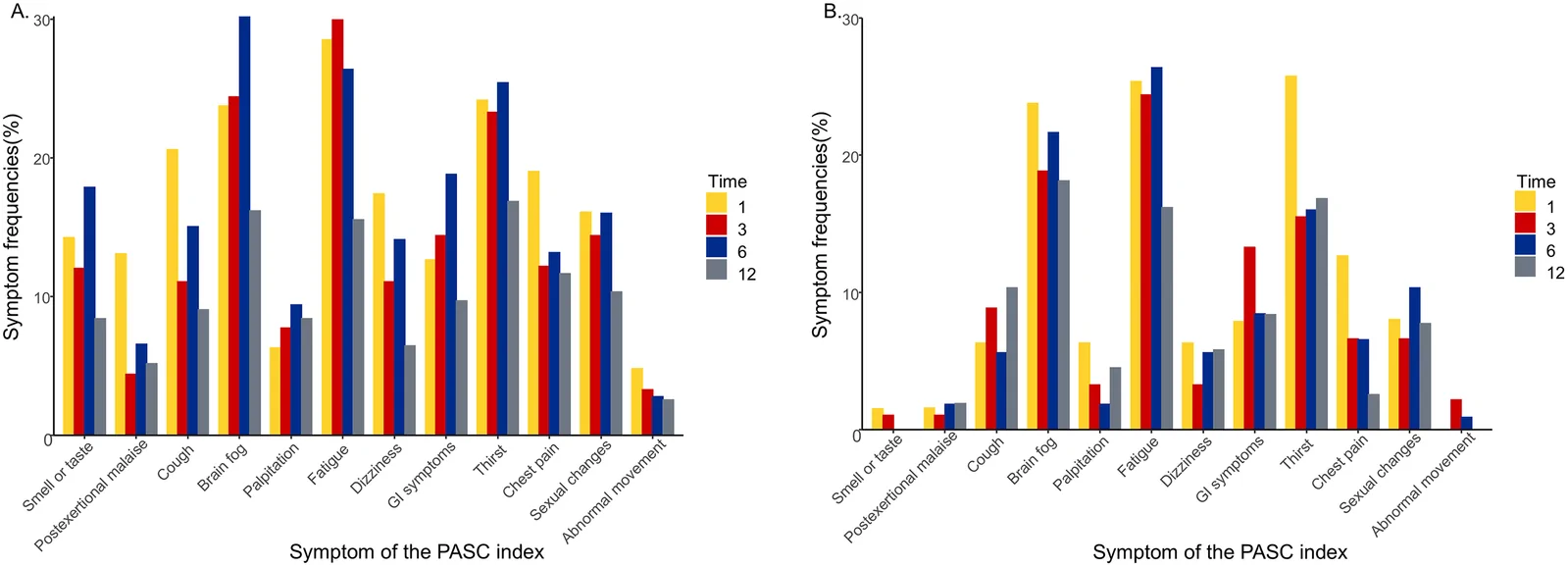
Time-dependent changes in the frequency of symptoms contributing to the PASC Index. (A) Frequency of symptoms among individuals with a PASC Index ≥12 across four time points. (B) Frequency of symptoms among individuals with a PASC Index <12 across four time points. a Each cell represents the symptom frequency (%).

When comparing the temporal changes in the individual symptoms comprising the PASC index, smell or taste changes and post-exertional malaise were relatively more frequent in the group with a PASC index ≥ 12 (Fig 3A). In contrast, symptoms such as brain fog, fatigue, and thirst persisted in both groups over time and were notably observed even among those with a PASC index < 12 (Fig 3B). In the complete-case sensitivity analysis using a more restrictive comparator definition, key symptoms, including fatigue, brain fog, and thirst, remained more frequent in the Long COVID group than in the non-Long COVID group (S2 Fig). However, these symptoms were not completely absent among non-Long COVID participants, suggesting that participants below the PASC index threshold may still have subthreshold symptoms.

**Fig 4:**
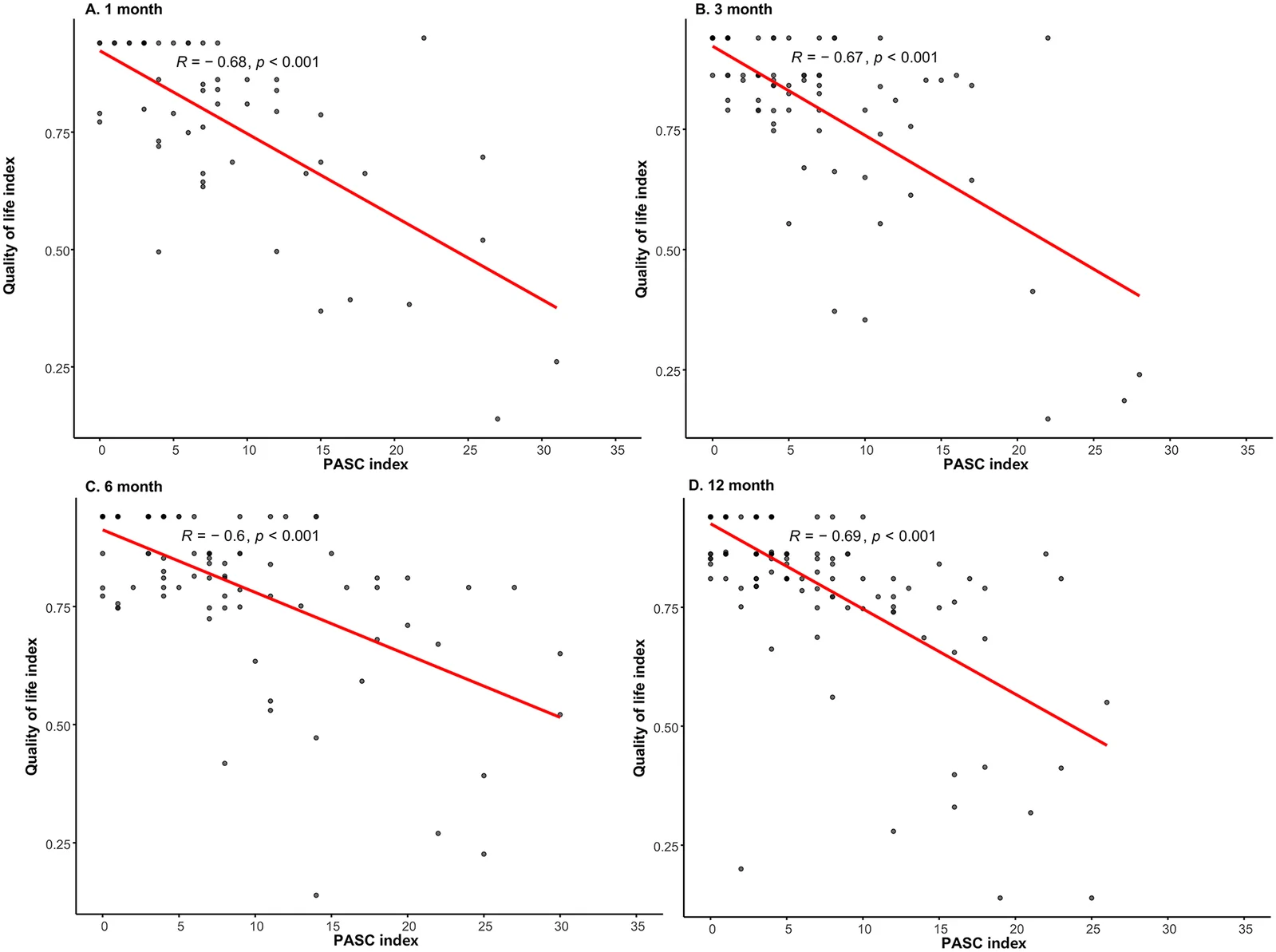
Correlation between PASC index and quality of life index. a Pearson correlation scatter plot of PASC Index vs. quality-of-life index. The red line represents the trend line. b Quality-of-life (QoL) index was assessed using the EQ-5D-5L (EuroQol 5-Dimension 5-Level) questionnaire. Scores were categorized as follows: severe (<0.4), moderate (<0.7), and mild (<0.9). Additional details are provided in S2 Table.

The PASC index showed a significant negative correlation with the quality of life (QoL) index (1 month: R = –0.68, P < 0.001; 3 months: R = –0.67, p < 0.001; 6 months: R = –0.60, p < 0.001; 12 months: R = –0.69, p < 0.001; Fig 4). QoL index were significantly different between the Long COVID group and the non-Long COVID group at all observed time points (S3 Fig).

## Discussion

In this prospective cohort study, we assessed the presence of Long COVID over time using the PASC index. During the one-year follow-up, 26% of participants had a PASC index ≥12 at least once. Although the prevalence was highest at one month and showed a tendency to decrease by twelve months, this decline was not statistically significant. Notably, individuals classified as having Long COVID tended to maintain similar PASC index levels over time. This suggests that, once participants developed a sufficiently complex and multifaceted symptom burden to meet the PASC index threshold, their overall symptom burden tended to persist, even though their threshold status could fluctuate across visits, particularly when scores were close to the cutoff.

Previous studies have reported varying estimates of Long COVID prevalence, depending on study design, population, and criteria used for defining Long COVID. The World Health Organization (WHO) estimates Long COVID prevalence at 10–20% beyond three months. Population-based studies reported rates of 12.7% in the Netherlands, 2.9% in the UK, and 6.6–10.3% in Scotland [8–10]. A study conducted in Mexico using the PASC index estimated the prevalence at 8.7% [11]. In the present study, the prevalence was comparatively higher, ranging from 16.7% to 27.0%. This discrepancy may be attributed to the clinical characteristics of the enrolled population, which included a higher burden of comorbidities known to be risk factors for Long COVID, as well as greater severity of acute COVID-19 [12,13].

A previous meta-analysis found that Long COVID symptoms can persist over time, with neuropsychiatric manifestations in particular showing prolonged courses [14]. In line with these findings, our study demonstrated that the PASC index in the Long COVID group remained relatively unchanged over time (Fig 2). Prior research has also identified fatigue and cognitive dysfunction as two of the most prevalent Long COVID symptoms [15]. Consistent with this observation, our results showed that fatigue, brain fog, and thirst were frequently reported by participants classified as Long COVID. However, these symptoms were also common among those in the non–Long COVID group (Fig 3), suggesting that individuals below the threshold (<12) may still experience Long COVID–related manifestations. Accordingly, comparisons using participants below the PASC index threshold as the reference group may have underestimated between-group differences. This limitation is inherent to threshold-based symptom classification, as individuals below the cutoff may still have possible or subthreshold Long COVID-related symptoms. This observation points to the possibility of “probable” Long COVID cases that are not fully captured by the PASC index cutoff. To more accurately identify and manage these cases, the development of additional diagnostic tools, including reliable biomarkers, is warranted.

Our findings indicate that the PASC index and Health-Related Quality of Life (HRQoL) were significantly correlated at all time points, and HRQoL differed markedly between the Long COVID and non-Long COVID groups. This finding aligns with previous research demonstrating an association between Long COVID and poorer quality of life [16–18], and underscores that the PASC index can help predict poor HRQoL outcomes, suggesting that the detrimental impact of long COVID on HRQoL may persist for up to one year post-infection.

Our study had several strengths. First of all, we defined Long COVID using the specific criterion of the PASC index. By evaluating the PASC index at multiple time points, we were able to examine time-dependent changes and delineate their kinetics. In addition, the prospective design minimized recall bias and enhanced the reliability of data collection. However, there are also several limitations. First, the relatively small, single-center tertiary-care cohort may have introduced selection bias and limited generalizability. Because our cohort included many medically complex patients, the observed prevalence of Long COVID should not be interpreted as a population-level estimate. Rather, our findings describe the longitudinal kinetics of the PASC index in a high-risk clinical cohort. Second, we only followed participants for up to 12 months, whereas Long COVID can reportedly persist beyond three years [2]. Larger, longer-term prospective studies using the PASC index are warranted. Third, response rates varied across follow-up, particularly in the early phase, resulting in incomplete data and potential differential non-response. Attrition analyses suggested differential non-response, and changes in respondent composition may have influenced the observed prevalence trajectory. Therefore, temporal changes in the proportion of participants meeting the PASC index threshold should be interpreted cautiously. However, a complete-case sensitivity analysis showed no significant within-person change in the PASC index from 1 to 12 months, supporting limited overall change among participants with complete follow-up, although attrition bias cannot be excluded. Fourth, because Long COVID was defined by meeting the PASC index threshold at least once during follow-up, some participants may have exceeded the threshold only transiently. Thus, symptom persistence should be interpreted as persistence of a multifaceted symptom burden rather than sustained threshold-level scores at every assessment. Lastly, participants in the Long COVID group had a higher burden of baseline risk factors, including older age, smoking, comorbidities, hospitalization, and COVID-19-related treatment, which may have contributed to persistent symptoms independently of Long COVID itself. Conversely, attrition analyses suggested that more comorbid and severely affected participants were less likely to contribute data at later assessments, potentially attenuating the observed symptom burden over time. These opposing sources of bias complicate interpretation; baseline imbalance may have contributed to the observed differences but may not fully explain the persistence of symptom burden during follow-up.

In conclusion, our study provides a longitudinal assessment of Long COVID using the PASC index over a 12-month period following acute SARS-CoV-2 infection. The proportion of participants meeting the cutoff for Long COVID (PASC index ≥12) remained clinically significant throughout the follow-up, underscoring the protracted and potentially chronic course of Long COVID. Larger-scale, multicenter studies with extended follow-up are warranted to validate these findings, refine diagnostic tools, and develop targeted interventions for individuals enduring persistent symptoms after SARS-CoV-2 infection.

## Supporting information

S1 Table The survey questions and scoring system consisting of the Post-acute Sequelae of SARS-CoV-2 Infection (PASC) index. (DOCX)

S2 Table Assessment and severity criteria for analysis. (DOCX)

S3 Table Effect sizes for pairwise within-person changes in the PASC index. (DOCX)

S4 Table Comparison of baseline characteristics according to response status at the 1-month follow-up. (DOCX)

S5 Table Comparison of baseline characteristics according to response status at the 3-month follow-up. (DOCX)

S6 Table Comparison of baseline characteristics according to response status at the 6-month follow-up. (DOCX)

S7 Table Comparison of baseline characteristics according to response status at the 12-month follow-up. (DOCX)

S1 Fig Longitudinal changes in the PASC index among participants with complete follow-up data. a Box plot: central line (median), box (IQR, interquartile range).
(TIF)

S2 Fig Time-dependent changes in the frequency of symptoms contributing to the PASC index in the complete-case sensitivity analysis. a Each bar represents the percentage of participants reporting the corresponding symptom (%).
(TIF)

S3 Fig Quality of life utility score over time. * The quality of life (QoL) utility score was assessed using the EQ-5D-5L (EuroQol 5-Dimension 5-Level) questionnaire. We defined scores below 0.4 as severe, scores below 0.7 as moderate, and scores below 0.9 as mild. Additional details are provided in the S2 Table * Pairwise Wilcoxon signed-rank tests with Bonferroni correction were applied to compare the differences in utility scores between long COVID and non-long COVID groups at each time point.
(TIF)

S1 Data Reference. (DOCX)

S1 File Supporting information. (CSV)
