# Supplementary material for: Kinetics of the PASC Index in Long COVID
Source: PLoS One. 2026 Sep 8;21(9):e0357670. doi: 10.1371/journal.pone.0357670 (PMC13552729; doi:10.1371/journal.pone.0357670)
Supplement: Supplementary file 1 [file pone.0357670.s001.docx]

**S1 Table.** The survey questions and scoring system consisting of the PASC index

| **Symptoms** | **Score** | **Survey questions or assessment criteria^a)^** |
| --- | --- | --- |
| Loss of or change in smell or taste change | 8 | “Have you had any recent changes or a loss in your sense of taste or smell?” |
| Post-exertional malaise | 7 | Patient-Reported Outcomes Measurement Information System (PROMIS) physical function 4a (T <=40) |
| Chronic cough | 4 | “Have you recently had a persistent or chronic cough?” |
| Brain fog | 3 | Quality of Life in Neurological Disorders (Neuro-QoL) Adult Applied Cognition-Executive Function (T <= 40) |
| Thirst | 3 | “Have you experienced persistent thirst recently?” |
| Palpitation | 2 | “Have you experienced symptoms of palpitations recently?” |
| Chest pain | 2 | Seattle Angina Questionnaire (SAQ)-7 (<75) |
| Fatigue | 1 | Fatigue Assessment Scale (FAS) (>=22) |
| Changes in sexual desires or capacity | 1 | “Have you recently experienced any changes in libido or sexual function?” |
| Dizziness | 1 | “Have you experienced dizziness recently?” |
| Gastrointestinal symptoms | 1 | When one or more symptoms, including nausea/vomiting, diarrhea, constipation, or abdominal pain, are observed |
| Abnormal movements | 1 | “Have you recently experienced any abnormal body movements?” |

^a)^ A symptom was considered present if the respondent answered affirmatively to the survey question or satisfied the assessment criteria
