# Supplementary material for: Kinetics of the PASC Index in Long COVID
Source: PLoS One. 2026 Sep 8;21(9):e0357670. doi: 10.1371/journal.pone.0357670 (PMC13552729; doi:10.1371/journal.pone.0357670)
Supplement: Supplementary file 2 [file pone.0357670.s002.docx]

**S2 Table.** Assessment and severity criteria for analysis

| **Assessment criteria** | **Definition** |
| --- | --- |
| PROMIS physical function 4a  (T <=40) | The PROMIS Physical Function Short Form 4a consists of four survey items, and raw scores were converted to T-scores based on a mean of 50 and a standard deviation of 10. A T-score below 40 was considered indicative of post-exertional malaise (PEM). |
| Neuro-QoL Adult Applied Cognition-Executive Function  (T <= 40) | The Neuro-QoL v2.0 Cognition Short Form was used, consisting of eight survey items related to perceived difficulties in mental functions such as planning, organizing, calculating, memory, and learning. Raw scores were converted to T-scores based on a mean of 50 and a standard deviation of 10. A T-score below 40 was considered indicative of brain fog. |
| Seattle Angina Questionnaire (SAQ-7) (<75) | SAQ-7 includes seven symptoms associated with chest pain and tightness. Chest pain was considered present with a score below 75. |
| Fatigue Assessment Scale (FAS) (>=22) | FAS is a 10-item self-report scale evaluating symptoms of chronic fatigue. Fatigue symptoms were considered present, with a score of 22 or above. |
| EuroQol (quality of life)  5-Dimension 5-Level (Eq-5d-5l) | The EQ-5D-5L is a tool designed to measure health-related quality of life across five dimensions, each representing a key aspect of health. These five dimensions are: mobility, self-care, usual activities, pain/discomfort, anxiety/depression. For each of these five dimensions, respondents select from five levels of severity. The utility score was calculated based on the five-digit code, which evaluates the five dimensions of EQ-5D-5L on a five-level scale, applying the weights derived from the South Korean value set.^1^ The utility score below 0.4 were defined as severe, those below 0.7 as moderate, and those below 0.9 as mild. |
