# Supplementary material for: Kinetics of the PASC Index in Long COVID
Source: PLoS One. 2026 Sep 8;21(9):e0357670. doi: 10.1371/journal.pone.0357670 (PMC13552729; doi:10.1371/journal.pone.0357670)
Supplement: Supplementary file 3 [file pone.0357670.s003.docx]

**S3 Table.** Effect sizes for pairwise within-person changes in the PASC index

| **Time point comparison** | **Long COVID** | **Non-long COVID** |
| --- | --- | --- |
| 1 vs 3 months | -0.46 | -0.11 |
| 3 vs 6 months | 0.21 | 0.06 |
| 6 vs 12 months | 0.02 | -0.15 |
| 1 vs 12 months | -0.22 | -0.15 |

Values are matched-pairs rank-biserial correlations for paired within-person comparisons of the PASC index between follow-up time points.
