# Supplementary material for: Kinetics of the PASC Index in Long COVID
Source: PLoS One. 2026 Sep 8;21(9):e0357670. doi: 10.1371/journal.pone.0357670 (PMC13552729; doi:10.1371/journal.pone.0357670)
Supplement: Supplementary file 4 [file pone.0357670.s004.docx]

**S4 Table.** Comparison of baseline characteristics according to response status at the 1-month follow-up

| **Characteristics** | **Responder**  **(n=63)** | **Non-responder**  **(n=120)** | **p-value** |
| --- | --- | --- | --- |
| Age (years), median (IQR) | 55.0 (33.0–66.0) | 43.0 (34.5–58.5) | 0.10 |
| Male gender | 28 (44.4) | 42 (35.0) | 0.26 |
| Obesity^a^ | 13 (21.0) | 30 (25.0) | 0.59 |
| Number of infections |  |  | 0.003 |
| 1 | 41 (66.1) | 103 (86.6) |  |
| 2 | 20 (32.3) | 15 (12.6) |  |
| 3 | 1 (1.6) | 1 (0.8) |  |
| Vaccination status^b^ |  |  | 0.58 |
| Unvaccinated | 3 (4.8) | 10 (8.4) |  |
| Partially vaccinated | 9 (14.3) | 13 (10.9) |  |
| Fully vaccinated | 51 (81.0) | 96 (80.7) |  |
| Smoking | 20 (32.3) | 24 (20.0) | 0.071 |
| Charlson comorbidity index, median (IQR) | 3.0 (0.0–5.0) | 0.0 (0.0–3.0) | <0.001 |
| Underlying disease |  |  |  |
| COPD | 1 (1.6) | 5 (4.2) | 0.67 |
| Asthma | 2 (3.2) | 2 (1.7) | 0.61 |
| Diabetes mellitus | 16 (25.8) | 21 (17.5) | 0.24 |
| CKD | 14 (22.6) | 13 (10.8) | 0.047 |
| Chronic liver diseases | 2 (3.2) | 3 (2.5) | >0.999 |
| Solid tumor | 13 (21.0) | 11 (9.2) | 0.04 |
| Hematologic malignancy | 5 (8.1) | 4 (3.3) | 0.28 |
| Hematopoietic stem cell transplant | 2 (3.2) | 2 (1.7) | 0.61 |
| Solid organ transplant | 15 (24.2) | 14 (11.7) | 0.03 |
| Mental health problems | 1 (1.6) | 5 (4.2) | 0.67 |
| Hospitalization for COVID-19 | 33 (53.2) | 37 (30.8) | 0.004 |
| Hospital treatment |  |  |  |
| Oxygen therapy | 18 (29.0) | 23 (19.2) | 0.14 |
| Steroid | 17 (27.4) | 27 (22.5) | 0.47 |
| Remdesivir | 34 (54.8) | 37 (30.8) | 0.002 |
| Tocilizumab | 4 (6.5) | 6 (5.0) | 0.74 |
| Baricitinib | 2(3.2) | 7 (5.8) | 0.72 |

Abbreviation: CKD, Chronic kidney disease; COPD, Chronic obstructive pulmonary disease; IQR, Interquartile range; PASC, Post-acute sequelae of SARS-CoV-2 infection

^a^Obesity defined as body mass index > 25 kg/m².

^b^Vaccination status defined as follows: unvaccinated (0 doses), partially vaccinated (1–2 doses), fully vaccinated (≥3 doses).
