# Supplementary material for: Kinetics of the PASC Index in Long COVID
Source: PLoS One. 2026 Sep 8;21(9):e0357670. doi: 10.1371/journal.pone.0357670 (PMC13552729; doi:10.1371/journal.pone.0357670)
Supplement: Supplementary file 5 [file pone.0357670.s005.docx]

**S5 Table.** Comparison of baseline characteristics according to response status at the 3-month follow-up

| **Characteristics** | **Responder**  **(n=90)** | **Non-responder**  **(n=93)** | **p-value** |
| --- | --- | --- | --- |
| Age (years), median (IQR) | 49.0 (33.0–66.0) | 46.0 (36.0–58.0) | 0.52 |
| Male gender | 32 (35.6) | 38 (40.9) | 0.54 |
| Obesity^a^ | 22 (24.7) | 21 (22.6) | 0.86 |
| Number of infections |  |  | <0.001 |
| 1 | 59 (67.0) | 85 (91.4) |  |
| 2 | 27 (30.7) | 8 (8.6) |  |
| 3 | 2 (2.3) | 0 (0.0) |  |
| Vaccination status^b^ |  |  | 0.78 |
| Unvaccinated | 5 (5.6) | 8 (8.6) |  |
| Partially vaccinated | 11 (12.4) | 11 (11.8) |  |
| Fully vaccinated | 73 (82.0) | 74 (79.6) |  |
| Smoking | 22 (24.7) | 22 (23.7) | >0.999 |
| Charlson comorbidity index, median (IQR) | 2.0 (0.0–4.0) | 0.0 (0.0–3.0) | 0.04 |
| Underlying disease |  |  |  |
| COPD | 2 (2.2) | 4 (4.3) | 0.68 |
| Asthma | 2 (2.2) | 2 (2.2) | >0.999 |
| Diabetes mellitus | 20 (22.5) | 17 (18.3) | 0.58 |
| CKD | 15 (16.9) | 12 (12.9) | 0.53 |
| Chronic liver diseases | 2 (2.2) | 3 (3.2) | >0.999 |
| Solid tumor | 16 (18.0) | 8 (8.6) | 0.08 |
| Hematologic malignancy | 6 (6.7) | 3 (3.2) | 0.32 |
| Hematopoietic stem cell transplant | 2 (2.2) | 2 (2.2) | >0.999 |
| Solid organ transplant | 14 (15.7) | 15 (16.1) | >0.999 |
| Mental health problems | 2 (2.3) | 4 (4.3) | 0.68 |
| Hospitalization for COVID-19 | 38 (42.7) | 32 (34.4) | 0.29 |
| Hospital treatment |  |  |  |
| Oxygen therapy | 21 (23.6) | 20 (21.5) | 0.86 |
| Steroid | 24 (27.0) | 20 (21.5) | 0.49 |
| Remdesivir | 39 (43.8) | 32 (34.4) | 0.23 |
| Tocilizumab | 4 (4.5) | 6 (6.5) | 0.75 |
| Baricitinib | 5 (5.6) | 4 (4.3) | 0.74 |

Abbreviation: CKD, Chronic kidney disease; COPD, Chronic obstructive pulmonary disease; IQR, Interquartile range; PASC, Post-acute sequelae of SARS-CoV-2 infection

^a^Obesity defined as body mass index > 25 kg/m².

^b^Vaccination status defined as follows: unvaccinated (0 doses), partially vaccinated (1–2 doses), fully vaccinated (≥3 doses).
