# Supplementary material for: Kinetics of the PASC Index in Long COVID
Source: PLoS One. 2026 Sep 8;21(9):e0357670. doi: 10.1371/journal.pone.0357670 (PMC13552729; doi:10.1371/journal.pone.0357670)
Supplement: Supplementary file 6 [file pone.0357670.s006.docx]

**S6 Table.** Comparison of baseline characteristics according to response status at the 6-month follow-up

| **Characteristics** | **Responder**  **(n=106)** | **Non-responder**  **(n=77)** | **p-value** |
| --- | --- | --- | --- |
| Age (years), median (IQR) | 52.0 (34.0–64.0) | 43.0 (35.0–56.0) | 0.17 |
| Male gender | 41 (38.7) | 29 (37.7) | >0.999 |
| Obesity^a^ | 21 (20.0) | 22 (28.6) | 0.22 |
| Number of infections |  |  | 0.003 |
| 1 | 75 (71.4) | 69 (90.8) |  |
| 2 | 28 (26.7) | 7 (9.2) |  |
| 3 | 2 (1.9) | 0 (0.0) |  |
| Vaccination status^b^ |  |  | 0.69 |
| Unvaccinated | 6 (5.7) | 7 (9.2) |  |
| Partially vaccinated | 13 (12.3) | 9 (11.8) |  |
| Fully vaccinated | 87 (82.1) | 60 (78.9) |  |
| Smoking | 28 (26.7) | 16 (20.8) | 0.39 |
| Charlson comorbidity index, median (IQR) | 2.0 (0.0–4.0) | 0.0 (0.0–3.0) | 0.02 |
| Underlying disease |  |  |  |
| COPD | 5 (4.8) | 1 (1.3) | 0.40 |
| Asthma | 2 (1.9) | 2 (2.6) | >0.999 |
| Diabetes mellitus | 23 (21.9) | 14 (18.2) | 0.58 |
| CKD | 19 (18.1) | 8 (10.4) | 0.21 |
| Chronic liver diseases | 3 (2.9) | 2 (2.6) | >0.999 |
| Solid tumor | 13 (12.4) | 11 (14.3) | 0.83 |
| Hematologic malignancy | 7 (6.7) | 2 (2.6) | 0.31 |
| Hematopoietic stem cell transplant | 3 (2.9) | 1 (1.3) | 0.64 |
| Solid organ transplant | 19 (18.1) | 10 (13.0) | 0.42 |
| Mental health problems | 3 (2.9) | 3 (3.9) | 0.70 |
| Hospitalization for COVID-19 | 50 (47.6) | 20 (26.0) | 0.003 |
| Hospital treatment |  |  |  |
| Oxygen therapy | 32 (30.5) | 9 (11.7) | 0.004 |
| Steroid | 36 (34.3) | 8 (10.4) | <0.001 |
| Remdesivir | 51 (48.6) | 20 (26.0) | 0.002 |
| Tocilizumab | 7 (6.7) | 3 (3.9) | 0.52 |
| Baricitinib | 6 (5.7) | 3 (3.9) | 0.74 |

Abbreviation: CKD, Chronic kidney disease; COPD, Chronic obstructive pulmonary disease; IQR, Interquartile range; PASC, Post-acute sequelae of SARS-CoV-2 infection

^a^Obesity defined as body mass index > 25 kg/m².

^b^Vaccination status defined as follows: unvaccinated (0 doses), partially vaccinated (1–2 doses), fully vaccinated (≥3 doses).
