# Supplementary material for: Kinetics of the PASC Index in Long COVID
Source: PLoS One. 2026 Sep 8;21(9):e0357670. doi: 10.1371/journal.pone.0357670 (PMC13552729; doi:10.1371/journal.pone.0357670)
Supplement: Supplementary file 7 [file pone.0357670.s007.docx]

**S7 Table.** Comparison of baseline characteristics according to response status at the 12-month follow-up

| **Characteristics** | **Responder**  **(n=154)** | **Non-responder**  **(n=29)** | **p-value** |
| --- | --- | --- | --- |
| Age (years), median (IQR) | 42.5 (34.0–60.0) | 63.0 (55.0–67.0) | <0.001 |
| Male gender | 52 (33.8) | 18 (62.1) | 0.006 |
| Obesity^a^ | 38 (24.8) | 5 (17.2) | 0.48 |
| Number of infections |  |  | 0.855 |
| 1 | 122 (79.7) | 22 (78.6) |  |
| 2 | 29 (19.0) | 6 (21.4) |  |
| 3 | 2 (1.3) | 0 (0.0) |  |
| Vaccination status^b^ |  |  | >0.999 |
| Unvaccinated | 11 (7.1) | 2 (7.1) |  |
| Partially vaccinated | 19 (12.3) | 3 (10.7) |  |
| Fully vaccinated | 124 (80.5) | 23 (82.1) |  |
| Smoking | 33 (21.6) | 11 (37.9) | 0.10 |
| Charlson comorbidity index, median (IQR) | 0.0 (0.0–3.0) | 4.0 (3.0–5.0) | <0.001 |
| Underlying disease |  |  |  |
| COPD | 4 (2.6) | 2 (6.9) | 0.24 |
| Asthma | 2 (1.3) | 2 (6.9) | 0.12 |
| Diabetes mellitus | 26 (17.0) | 11 (37.9) | 0.02 |
| CKD | 22 (14.4) | 5 (17.2) | 0.78 |
| Chronic liver diseases | 3 (2.0) | 2 (6.9) | 0.18 |
| Solid tumor | 13 (8.5) | 11 (37.9) | <0.001 |
| Hematologic malignancy | 8 (5.2) | 1 (3.4) | >0.999 |
| Hematopoietic stem cell transplant | 3 (2.0) | 1 (3.4) | 0.50 |
| Solid organ transplant | 23 (15.0) | 6 (20.7) | 0.42 |
| Mental health problems | 4 (2.6) | 2 (6.9) | 0.25 |
| Hospitalization for COVID-19 | 51 (33.3) | 19 (65.5) | 0.002 |
| Hospital treatment |  |  |  |
| Oxygen therapy | 33 (21.6) | 8 (27.6) | 0.47 |
| Steroid | 38 (24.8) | 6 (20.7) | 0.81 |
| Remdesivir | 52 (34.0) | 19 (65.5) | 0.002 |
| Tocilizumab | 8 (5.2) | 2 (6.9) | 0.66 |
| Baricitinib | 9 (5.9) | 0 (0.0) | 0.36 |

Abbreviation: CKD, Chronic kidney disease; COPD, Chronic obstructive pulmonary disease; IQR, Interquartile range; PASC, Post-acute sequelae of SARS-CoV-2 infection

^a^Obesity defined as body mass index > 25 kg/m².

^b^Vaccination status defined as follows: unvaccinated (0 doses), partially vaccinated (1–2 doses), fully vaccinated (≥3 doses).
