# Supplementary material for: Kinetics of the PASC Index in Long COVID
Source: PLoS One. 2026 Sep 8;21(9):e0357670. doi: 10.1371/journal.pone.0357670 (PMC13552729; doi:10.1371/journal.pone.0357670)
Supplement: Supplementary file 8 [file pone.0357670.s011.docx]

**S1 Reference.**

1.	Kim SH, Ahn J, Ock M, et al. The EQ-5D-5L valuation study in Korea. Qual Life Res. 2016;25(7):1845-1852. doi:10.1007/s11136-015-1205-2\
