# Supplementary material for: Kinetics of the PASC Index in Long COVID
Source: PLoS One. 2026 Sep 8;21(9):e0357670. doi: 10.1371/journal.pone.0357670 (PMC13552729; doi:10.1371/journal.pone.0357670)
Supplement: Supplementary file 9 [file pone.0357670.s014.docx]

**Dr. Kokouvi Kassegne**

Acadenuc Editor

*PLOS ONE*

**July 28, 2026**

**Manuscript No: PONE-D-25-23610**

Dear Dr. Kassegne

Thank you for the helpful comments on our manuscript. Below we provide our responses to these comments.

**Journal requirements**

1. Please ensure that your manuscript meets PLOS ONE's style requirements, including those for file naming.

🡪 Thank you for your comment. We have carefully reviewed the manuscript to ensure that it meets PLOS ONE’s style requirements, including the requirements for file naming.

2. Thank you for stating in your Funding Statement: “This work was supported by part of Korea Health Technology Research and Development Project through the Korea Health Industry Development Institute (KHIDI), which is funded by the National Institute of Infectious Diseases, National Institute of Health, Republic of Korea (grant No. HD22C2045).?” Please provide an amended statement that declares *all* the funding or sources of support (whether external or internal to your organization) received during this study, as detailed online in our guide for authors at http://journals.plos.org/plosone/s/submit-now. Please also include the statement “There was no additional external funding received for this study.” in your updated Funding Statement. Please include your amended Funding Statement within your cover letter. We will change the online submission form on your behalf.

🡪 Thank you for your comment. We have amended the Funding Statement to declare all funding and sources of support received during this study. The amended Funding Statement has been included in the cover letter, as requested.

**Funding/Support**

We corrected the grant information to reflect the funding source that supported this study. This research was supported by the National Institute of Health (NIH), Republic of Korea, research project (2026-ER1901-00). There was no additional external funding received for this study. No additional internal funding or sources of support were received for this study.

3. Thank you for stating the following financial disclosure: “This work was supported by part of the Korea Health Technology Research and Development Project through the Korea Health Industry Development Institute (KHIDI), which is funded by the National Institute of Infectious Diseases, National Institute of Health, Republic of Korea (grant No. HD22C2045).” Please state what role the funders took in the study. If the funders had no role, please state: "The funders had no role in study design, data collection and analysis, decision to publish, or preparation of the manuscript. "If this statement is not correct you must amend it as needed. Please include this amended Role of Funder statement in your cover letter; we will change the online submission form on your behalf.

🡪 Thank you for your comment. We have amended the Role of Funder statement and included it in the cover letter, as requested.

**Role of the Funder**

The funders had no role in study design, data collection and analysis, decision to publish, or preparation of the manuscript.

4. In the online submission form, you indicated that [Any inquiries regarding supporting data availability of this study should be directed to the corresponding author.]. All PLOS journals now require all data underlying the findings described in their manuscript to be freely available to other researchers, either 1. In a public repository, 2. Within the manuscript itself, or 3. Uploaded as supplementary information. This policy applies to all data except where public deposition would breach compliance with the protocol approved by your research ethics board. If your data cannot be made publicly available for ethical or legal reasons (e.g., public availability would compromise patient privacy), please explain your reasons on resubmission and your exemption request will be escalated for approval.

🡪 Thank you for your comment. We have amended the Data Availability Statement in accordance with PLOS ONE’s data availability policy. The data underlying the findings of this study are now provided as Supporting Information files.

**Data availability statement**

All relevant data underlying the findings of this study are within the manuscript and its Supporting Information files.

**Editor comment**

**Major comment 1: Response rate bias threatens validity. Only 34% completed the 1-month assessment versus 84% at 12 months. This inverse pattern is unusual and suggests selective non-response (e.g., asymptomatic individuals dropping out early). The authors must provide attrition analyses comparing baseline characteristics of responders vs. non-responders at each time point.**

🡪 We thank the Editor for this important comment. First of all, the inverse pattern reflects the first enrollment timing of the participants in the study. Of a total of 183 patients, the first questionnaire was performed as follows; 63 (34.4%) at 1 month, 38 (20.8%) at 3 months, 22 (12.0%) at 6 months, and 60 (32.8%) at 12 months. Despite of this, we agree that the low and varying response rates across follow-up time points may introduce attrition bias and affect the interpretation of longitudinal prevalence estimates. To address this concern, we performed attrition analyses comparing baseline characteristics between responders and non-responders at each follow-up time point. Responders were defined as participants with a non-missing PASC index score at the corresponding assessment.

The attrition analyses showed that response patterns differed according to baseline clinical characteristics. At the 1-month assessment, responders had a greater burden of comorbidities and more severe acute COVID-19 than non-responders, including higher Charlson comorbidity index scores and higher proportions of chronic kidney disease, solid tumor, solid organ transplantation, hospitalization for COVID-19, and remdesivir treatment. Repeated SARS-CoV-2 infections were also more common among 1-month responders. Conversely, at the 12-month assessment, non-responders were older, more frequently male, and had higher Charlson comorbidity index scores and higher proportions of diabetes mellitus, solid tumor, hospitalization for COVID-19, and remdesivir treatment.

These findings indicate that missingness was unlikely to be completely random. In addition, many participants who contributed data at 12 months had not responded at the 1-month assessment, suggesting that 12-month respondents differed in composition from those assessed earlier. Therefore, the observed decline in the proportion of participants meeting the PASC index threshold may partly reflect differential non-response and changes in respondent composition, rather than natural symptom resolution alone.

To further examine whether this pattern was driven solely by changes in respondent composition, we performed a complete-case sensitivity analysis restricted to participants who completed all four follow-up assessments. Pairwise within-person comparisons using the Wilcoxon signed-rank test with Bonferroni correction showed no significant differences between 1 and 3 months, 3 and 6 months, 6 and 12 months, or 1 and 12 months (all adjusted p > 0.999), suggesting that symptom burden, as measured by the PASC index, remained persistent over 12 months among participants with complete longitudinal follow-up (S1 Fig).

We have added the attrition analyses as S4–7 Table and the complete-case sensitivity analysis to the Results section. We also revised the Limitations section to explicitly acknowledge this potential bias and its effect on prevalence estimates. Accordingly, we have toned down the interpretation of prevalence kinetics and clarified that our findings describe observed symptom trajectories among respondents in this cohort rather than unbiased population-level prevalence estimates.

1) Revised) Methods, Line number 79–83

A total of 183 participants were enrolled. The distribution of participants according to the timing of their first completed questionnaire was as follows: 63 (34.4%) at 1 month, 38 (20.8%) at 3 months, 22 (12.0%) at 6 months, and 60 (32.8%) at 12 months after infection. The overall numbers of participants who completed the questionnaire at each follow-up assessment were 63 of 183 (34.4%) at 1 month, 90 of 183 (49.2%) at 3 months, 106 of 183 (57.9%) at 6 months, and 154 of 183 (84.2%) at 12 months.

2) Revised) Methods, Line number 110–115

To assess potential attrition bias, we compared baseline characteristics between responders and non-responders at each follow-up time point. Responders were defined as participants with a non-missing PASC index score at the corresponding assessment. We also performed a complete-case sensitivity analysis restricted to participants who completed all four follow-up assessments, applying the same paired Wilcoxon signed-rank testing framework.

3) Revised) Results, Line number 180–190

**Attrition and complete-case sensitivity analyses**

Attrition analyses showed differential response patterns across follow-up time points (S4–7 Tables). Early responders tended to have greater comorbidity burden and more severe acute COVID-19-related features, whereas 12-month non-responders were older and had greater comorbidity and severity profiles than 12-month responders.

In the complete-case sensitivity analysis restricted to participants who completed all four assessments (n = 39), pairwise within-person comparisons using the Wilcoxon signed-rank test showed no significant differences in the PASC index across adjacent time points or between 1 and 12 months. The raw p-values were 0.31 for 1 vs. 3 months, 0.97 for 3 vs. 6 months, 0.78 for 6 vs. 12 months, and 0.50 for 1 vs. 12 months (S1 Fig). After Bonferroni correction for multiple comparisons, all adjusted p-values were >0.999.

4) Revised) Table S4–7. S1 Fig.

**S1 Fig**. Longitudinal changes in the PASC index among participants with complete follow-up data.

^a^ Box plot: central line (median), box (IQR, interquartile range).

**S4 Table.** Comparison of baseline characteristics according to response status at the 1-month follow-up

| **Characteristics** | **Responder**  **(n=63)** | **Non-responder**  **(n=120)** | **p-value** |
| --- | --- | --- | --- |
| Age (years), median (IQR) | 55.0 (33.0–66.0) | 43.0 (34.5–58.5) | 0.10 |
| Male gender | 28 (44.4) | 42 (35.0) | 0.26 |
| Obesity^a^ | 13 (21.0) | 30 (25.0) | 0.59 |
| Number of infections |  |  | 0.003 |
| 1 | 41 (66.1) | 103 (86.6) |  |
| 2 | 20 (32.3) | 15 (12.6) |  |
| 3 | 1 (1.6) | 1 (0.8) |  |
| Vaccination status^b^ |  |  | 0.58 |
| Unvaccinated | 3 (4.8) | 10 (8.4) |  |
| Partially vaccinated | 9 (14.3) | 13 (10.9) |  |
| Fully vaccinated | 51 (81.0) | 96 (80.7) |  |
| Smoking | 20 (32.3) | 24 (20.0) | 0.071 |
| Charlson comorbidity index, median (IQR) | 3.0 (0.0–5.0) | 0.0 (0.0–3.0) | <0.001 |
| Underlying disease |  |  |  |
| COPD | 1 (1.6) | 5 (4.2) | 0.67 |
| Asthma | 2 (3.2) | 2 (1.7) | 0.61 |
| Diabetes mellitus | 16 (25.8) | 21 (17.5) | 0.24 |
| CKD | 14 (22.6) | 13 (10.8) | 0.047 |
| Chronic liver diseases | 2 (3.2) | 3 (2.5) | >0.999 |
| Solid tumor | 13 (21.0) | 11 (9.2) | 0.04 |
| Hematologic malignancy | 5 (8.1) | 4 (3.3) | 0.28 |
| Hematopoietic stem cell transplant | 2 (3.2) | 2 (1.7) | 0.61 |
| Solid organ transplant | 15 (24.2) | 14 (11.7) | 0.03 |
| Mental health problems | 1 (1.6) | 5 (4.2) | 0.67 |
| Hospitalization for COVID-19 | 33 (53.2) | 37 (30.8) | 0.004 |
| Hospital treatment |  |  |  |
| Oxygen therapy | 18 (29.0) | 23 (19.2) | 0.14 |
| Steroid | 17 (27.4) | 27 (22.5) | 0.47 |
| Remdesivir | 34 (54.8) | 37 (30.8) | 0.002 |
| Tocilizumab | 4 (6.5) | 6 (5.0) | 0.74 |
| Baricitinib | 2(3.2) | 7 (5.8) | 0.72 |

Abbreviation: CKD, Chronic kidney disease; COPD, Chronic obstructive pulmonary disease; IQR, Interquartile range; PASC, Post-acute sequelae of SARS-CoV-2 infection

^a^Obesity defined as body mass index > 25 kg/m².

^b^Vaccination status defined as follows: unvaccinated (0 doses), partially vaccinated (1–2 doses), fully vaccinated (≥3 doses).

**S5 Table.** Comparison of baseline characteristics according to response status at the 3-month follow-up

| **Characteristics** | **Responder**  **(n=90)** | **Non-responder**  **(n=93)** | **p-value** |
| --- | --- | --- | --- |
| Age (years), median (IQR) | 49.0 (33.0–66.0) | 46.0 (36.0–58.0) | 0.52 |
| Male gender | 32 (35.6) | 38 (40.9) | 0.54 |
| Obesity^a^ | 22 (24.7) | 21 (22.6) | 0.86 |
| Number of infections |  |  | <0.001 |
| 1 | 59 (67.0) | 85 (91.4) |  |
| 2 | 27 (30.7) | 8 (8.6) |  |
| 3 | 2 (2.3) | 0 (0.0) |  |
| Vaccination status^b^ |  |  | 0.78 |
| Unvaccinated | 5 (5.6) | 8 (8.6) |  |
| Partially vaccinated | 11 (12.4) | 11 (11.8) |  |
| Fully vaccinated | 73 (82.0) | 74 (79.6) |  |
| Smoking | 22 (24.7) | 22 (23.7) | >0.999 |
| Charlson comorbidity index, median (IQR) | 2.0 (0.0–4.0) | 0.0 (0.0–3.0) | 0.04 |
| Underlying disease |  |  |  |
| COPD | 2 (2.2) | 4 (4.3) | 0.68 |
| Asthma | 2 (2.2) | 2 (2.2) | >0.999 |
| Diabetes mellitus | 20 (22.5) | 17 (18.3) | 0.58 |
| CKD | 15 (16.9) | 12 (12.9) | 0.53 |
| Chronic liver diseases | 2 (2.2) | 3 (3.2) | >0.999 |
| Solid tumor | 16 (18.0) | 8 (8.6) | 0.08 |
| Hematologic malignancy | 6 (6.7) | 3 (3.2) | 0.32 |
| Hematopoietic stem cell transplant | 2 (2.2) | 2 (2.2) | >0.999 |
| Solid organ transplant | 14 (15.7) | 15 (16.1) | >0.999 |
| Mental health problems | 2 (2.3) | 4 (4.3) | 0.68 |
| Hospitalization for COVID-19 | 38 (42.7) | 32 (34.4) | 0.29 |
| Hospital treatment |  |  |  |
| Oxygen therapy | 21 (23.6) | 20 (21.5) | 0.86 |
| Steroid | 24 (27.0) | 20 (21.5) | 0.49 |
| Remdesivir | 39 (43.8) | 32 (34.4) | 0.23 |
| Tocilizumab | 4 (4.5) | 6 (6.5) | 0.75 |
| Baricitinib | 5 (5.6) | 4 (4.3) | 0.74 |

Abbreviation: CKD, Chronic kidney disease; COPD, Chronic obstructive pulmonary disease; IQR, Interquartile range; PASC, Post-acute sequelae of SARS-CoV-2 infection

^a^Obesity defined as body mass index > 25 kg/m².

^b^Vaccination status defined as follows: unvaccinated (0 doses), partially vaccinated (1–2 doses), fully vaccinated (≥3 doses).

**S6 Table.** Comparison of baseline characteristics according to response status at the 6-month follow-up

| **Characteristics** | **Responder**  **(n=106)** | **Non-responder**  **(n=77)** | **p-value** |
| --- | --- | --- | --- |
| Age (years), median (IQR) | 52.0 (34.0–64.0) | 43.0 (35.0–56.0) | 0.17 |
| Male gender | 41 (38.7) | 29 (37.7) | >0.999 |
| Obesity^a^ | 21 (20.0) | 22 (28.6) | 0.22 |
| Number of infections |  |  | 0.003 |
| 1 | 75 (71.4) | 69 (90.8) |  |
| 2 | 28 (26.7) | 7 (9.2) |  |
| 3 | 2 (1.9) | 0 (0.0) |  |
| Vaccination status^b^ |  |  | 0.69 |
| Unvaccinated | 6 (5.7) | 7 (9.2) |  |
| Partially vaccinated | 13 (12.3) | 9 (11.8) |  |
| Fully vaccinated | 87 (82.1) | 60 (78.9) |  |
| Smoking | 28 (26.7) | 16 (20.8) | 0.39 |
| Charlson comorbidity index, median (IQR) | 2.0 (0.0–4.0) | 0.0 (0.0–3.0) | 0.02 |
| Underlying disease |  |  |  |
| COPD | 5 (4.8) | 1 (1.3) | 0.40 |
| Asthma | 2 (1.9) | 2 (2.6) | >0.999 |
| Diabetes mellitus | 23 (21.9) | 14 (18.2) | 0.58 |
| CKD | 19 (18.1) | 8 (10.4) | 0.21 |
| Chronic liver diseases | 3 (2.9) | 2 (2.6) | >0.999 |
| Solid tumor | 13 (12.4) | 11 (14.3) | 0.83 |
| Hematologic malignancy | 7 (6.7) | 2 (2.6) | 0.31 |
| Hematopoietic stem cell transplant | 3 (2.9) | 1 (1.3) | 0.64 |
| Solid organ transplant | 19 (18.1) | 10 (13.0) | 0.42 |
| Mental health problems | 3 (2.9) | 3 (3.9) | 0.70 |
| Hospitalization for COVID-19 | 50 (47.6) | 20 (26.0) | 0.003 |
| Hospital treatment |  |  |  |
| Oxygen therapy | 32 (30.5) | 9 (11.7) | 0.004 |
| Steroid | 36 (34.3) | 8 (10.4) | <0.001 |
| Remdesivir | 51 (48.6) | 20 (26.0) | 0.002 |
| Tocilizumab | 7 (6.7) | 3 (3.9) | 0.52 |
| Baricitinib | 6 (5.7) | 3 (3.9) | 0.74 |

Abbreviation: CKD, Chronic kidney disease; COPD, Chronic obstructive pulmonary disease; IQR, Interquartile range; PASC, Post-acute sequelae of SARS-CoV-2 infection

^a^Obesity defined as body mass index > 25 kg/m².

^b^Vaccination status defined as follows: unvaccinated (0 doses), partially vaccinated (1–2 doses), fully vaccinated (≥3 doses).

**S7 Table.** Comparison of baseline characteristics according to response status at the 12-month follow-up

| **Characteristics** | **Responder**  **(n=154)** | **Non-responder**  **(n=29)** | **p-value** |
| --- | --- | --- | --- |
| Age (years), median (IQR) | 42.5 (34.0–60.0) | 63.0 (55.0–67.0) | <0.001 |
| Male gender | 52 (33.8) | 18 (62.1) | 0.006 |
| Obesity^a^ | 38 (24.8) | 5 (17.2) | 0.48 |
| Number of infections |  |  | 0.855 |
| 1 | 122 (79.7) | 22 (78.6) |  |
| 2 | 29 (19.0) | 6 (21.4) |  |
| 3 | 2 (1.3) | 0 (0.0) |  |
| Vaccination status^b^ |  |  | >0.999 |
| Unvaccinated | 11 (7.1) | 2 (7.1) |  |
| Partially vaccinated | 19 (12.3) | 3 (10.7) |  |
| Fully vaccinated | 124 (80.5) | 23 (82.1) |  |
| Smoking | 33 (21.6) | 11 (37.9) | 0.10 |
| Charlson comorbidity index, median (IQR) | 0.0 (0.0–3.0) | 4.0 (3.0–5.0) | <0.001 |
| Underlying disease |  |  |  |
| COPD | 4 (2.6) | 2 (6.9) | 0.24 |
| Asthma | 2 (1.3) | 2 (6.9) | 0.12 |
| Diabetes mellitus | 26 (17.0) | 11 (37.9) | 0.02 |
| CKD | 22 (14.4) | 5 (17.2) | 0.78 |
| Chronic liver diseases | 3 (2.0) | 2 (6.9) | 0.18 |
| Solid tumor | 13 (8.5) | 11 (37.9) | <0.001 |
| Hematologic malignancy | 8 (5.2) | 1 (3.4) | >0.999 |
| Hematopoietic stem cell transplant | 3 (2.0) | 1 (3.4) | 0.50 |
| Solid organ transplant | 23 (15.0) | 6 (20.7) | 0.42 |
| Mental health problems | 4 (2.6) | 2 (6.9) | 0.25 |
| Hospitalization for COVID-19 | 51 (33.3) | 19 (65.5) | 0.002 |
| Hospital treatment |  |  |  |
| Oxygen therapy | 33 (21.6) | 8 (27.6) | 0.47 |
| Steroid | 38 (24.8) | 6 (20.7) | 0.81 |
| Remdesivir | 52 (34.0) | 19 (65.5) | 0.002 |
| Tocilizumab | 8 (5.2) | 2 (6.9) | 0.66 |
| Baricitinib | 9 (5.9) | 0 (0.0) | 0.36 |

Abbreviation: CKD, Chronic kidney disease; COPD, Chronic obstructive pulmonary disease; IQR, Interquartile range; PASC, Post-acute sequelae of SARS-CoV-2 infection

^a^Obesity defined as body mass index > 25 kg/m².

^b^Vaccination status defined as follows: unvaccinated (0 doses), partially vaccinated (1–2 doses), fully vaccinated (≥3 doses).

5) Revised) Discussion, Line number 274–282

Third, response rates varied across follow-up, particularly in the early phase, resulting in incomplete data and potential differential non-response. Attrition analyses suggested differential non-response, and changes in respondent composition may have influenced the observed prevalence trajectory. Therefore, temporal changes in the proportion of participants meeting the PASC index threshold should be interpreted cautiously. However, a complete-case sensitivity analysis showed no significant within-person change in the PASC index from 1 to 12 months, supporting limited overall change among participants with complete follow-up, although attrition bias cannot be excluded.

**Major comment 2: The "non-PASC" group contains probable PASC cases. Figure 3B shows fatigue, brain fog, and thirst are common in the <12 group. Using "never met criteria" as the comparator dilutes effect sizes. Consider sensitivity analyses defining non-PASC as consistently ≤11 across all time points.**

🡪 We thank the Editor for this important comment. We agree that participants with a PASC index below 12 may still have clinically relevant symptoms and should not be interpreted as completely asymptomatic controls. Symptoms such as fatigue, brain fog, and thirst are relatively non-specific and may occur without the total weighted PASC index reaching the diagnostic threshold. Therefore, the below-threshold group may include participants with subthreshold or probable PASC manifestations, which could dilute between-group differences.

To address this issue, we performed a complete-case sensitivity analysis using a more restrictive comparator definition. In this analysis, the non-long COVID group was defined as participants who completed all four follow-up assessments and had a PASC index ≤11 at every time point. Key symptoms, including fatigue, brain fog, and thirst, remained more frequent in the Long COVID group than in this non-Long COVID group. However, these symptoms were not completely absent among non-Long COVID participants, supporting the Editor’s concern that participants below the threshold may still have subthreshold symptoms. Overall, the findings were consistent with the main analysis, suggesting that our results were not driven solely by comparator misclassification, while still acknowledging the limitations of using a threshold-based classification.

This issue is also consistent with the way the RECOVER PASC index has been framed. The original RECOVER PASC index was proposed as an initial symptom-based classification framework rather than a definitive boundary separating symptomatic from asymptomatic individuals. Individuals who do not meet the score threshold may still require consideration of additional clinical or symptom data. Similarly, the updated RECOVER-Adult Long COVID Research Index introduced a “possible Long COVID” category, further supporting the concept that individuals below the threshold may still have subthreshold or probable Long COVID-related symptoms.

We have added this sensitivity analysis to the Results and added corresponding descriptions to the Methods and Discussion.

1) Revised) Methods, Line number 115–120

Separately, to address the possibility that participants below the PASC index threshold may have included subthreshold or probable Long COVID cases, we performed an additional complete-case sensitivity analysis using a more restrictive comparator definition. In this analysis, the non-Long COVID group was defined as participants who completed all four follow-up assessments and had a PASC index ≤11 at every time point.

2) Revised) Results, Line number 197–202

In the complete-case sensitivity analysis using a more restrictive comparator definition, key symptoms, including fatigue, brain fog, and thirst, remained more frequent in the Long COVID group than in the non-Long COVID group (S2 Fig). However, these symptoms were not completely absent among non-Long COVID participants, suggesting that participants below the PASC index threshold may still have subthreshold symptoms.

3) Revised) Discussion, Line number 251–254

Accordingly, comparisons using participants below the PASC index threshold as the reference group may have underestimated between-group differences. This limitation is inherent to threshold-based symptom classification, as individuals below the cutoff may still have possible or subthreshold Long COVID-related symptoms.

**Major Comment 3. Statistical reporting inconsistency. Abstract states prevalence decline from 27% to 18% was non-significant (p=0.16), but Results reports a significant OR reduction at 3 months (p=0.04). Clarify which analysis is primary—GEE or pairwise comparisons.**

🡪 Thank you for the valuable comment. We agree that the original wording did not clearly distinguish the primary longitudinal contrast from additional time-specific comparisons. We have clarified the statistical reporting to distinguish the longitudinal GEE analysis of the binary PASC index threshold from the pairwise Wilcoxon signed-rank tests used for the continuous PASC index. Our primary contrast of interest for the prevalence of Long COVID was the change between 1 and 12 months, reflecting the 12-month trajectory of PASC classification.

1) Revised) Methods, Line number 99–105

The primary longitudinal analysis evaluated changes in the proportion of participants with a PASC index ≥12 over time using generalized estimating equations (GEE) with an autoregressive(1) working correlation structure. Time was modeled as a categorical variable, with the 1-month assessment as the reference. Within-person changes in the continuous PASC index were evaluated using pairwise Wilcoxon signed-rank tests with Bonferroni correction for multiple comparisons.

2) Revised) Results, Line number 152–157

The prevalence of Long COVID was 27.0% (17/63) at 1 month, 16.7% (15/90) at 3 months, 21.7% (23/106) at 6 months, and 17.5% (27/154) at 12 months (Fig 1). In the GEE analysis using 1 month as the reference, the difference at 12 months was not statistically significant (OR = 0.64, p = 0.16). Although the odds of meeting the PASC index threshold were significantly lower at 3 months (OR = 0.46, p = 0.04), this reduction was not sustained at 6 months (OR = 0.57, p = 0.13) or 12 months.

**Minor comment4: "Kinetics of PASC Index in Patients with PASC": suggest "Kinetics of the PASC Index in Long COVID.”**

🡪 We thank the Editor for this helpful suggestion. We agree that the original title was redundant and have revised the title accordingly.

1) Revised) Title

Kinetics of the PASC Index in Long COVID

2) Revised) Short title

**Short title:** PASC Index Kinetics

**Minor comment 5: Missing effect sizes. Provide Cohen's d or rank-biserial correlation for pairwise comparisons.**

🡪 We thank the Editor for this helpful suggestion. We have added matched-pairs rank-biserial correlations as the corresponding effect size measure. In the Long COVID group, the rank-biserial correlations were −0.46 for 1 vs. 3 months, +0.21 for 3 vs. 6 months, +0.02 for 6 vs. 12 months, and −0.22 for 1 vs. 12 months. In the non-Long COVID group, the corresponding values were −0.11, +0.06, −0.15, and −0.15. The largest value reflected a transient reduction at 3 months in the Long COVID group; importantly, the net change over the full follow-up (1 vs. 12 months) showed small effect sizes in both groups, consistent with minimal overall within-person change over the follow-up period rather than insufficient statistical power.

1) Revised) Methods, Line number 105–106

For pairwise within-person comparisons of the continuous PASC index, effect sizes were reported as matched-pairs rank-biserial correlations.

2) Revised) Results, Line number 171–175

Effect sizes for all pairwise within-person comparisons, expressed as matched-pairs rank-biserial correlations, are presented in S3 Table. The 1-to-12-month comparisons showed small effect sizes in both the Long COVID and non-Long COVID groups, consistent with minimal overall within-person change over the full follow-up period.

3) Revised) S3 Table

**S3 Table**. Effect sizes for pairwise within-person changes in the PASC index

| **Time point comparison** | **Long COVID** | **Non-long COVID** |
| --- | --- | --- |
| 1 vs 3 months | -0.46 | -0.11 |
| 3 vs 6 months | 0.21 | 0.06 |
| 6 vs 12 months | 0.02 | -0.15 |
| 1 vs 12 months | -0.22 | -0.15 |

Values are matched-pairs rank-biserial correlations for paired within-person comparisons of the PASC index between follow-up time points.

**Minor comment 6: Figure 2B label error. "PASC index ≤12" is all individuals below threshold; this describes the non-PASC group, not the caption's claim of "consistently ≤12 across all time points.”**

🡪 We thank the Editor for pointing out this labeling issue. We agree that the previous label could be confusing. To avoid ambiguity, we have revised the Figure 2 caption to use the predefined group labels, “Long COVID group” and “non-Long COVID group,” consistent with the definitions used throughout the manuscript.

1) Revised)

**Fig 2.** Temporal changes in the PASC index according to Long COVID status.
(A) Long COVID group. (B) Non-Long COVID group.

**Minor Comment 7. Data availability statement insufficient. "Direct inquiries to corresponding author" does not meet PLOS ONE's requirement for full data access. Please deposit anonymized data in a public repository with a DOI.**

🡪 We thank the Editor for this comment. Please refer to our response to Journal Requirement 4, where we have addressed the Data Availability Statement in accordance with PLOS ONE’s requirements.

**Reviewer #1 comment**

**Comment 1: Use consistent terminology, e.g., COVID or COVID-19.**

🡪 We thank the reviewer for this helpful comment. We have reviewed the manuscript and standardized terminology throughout. We now use “Long COVID” for the post-acute condition defined using the PASC index. We also standardized the capitalization of “Long COVID” throughout the manuscript and revised inconsistent expressions such as “COVID-19 infection” where appropriate.

**Comment 2: Full data should be made available as supplementary material.**

🡪 We thank the reviewer for this comment. Please refer to our response to Journal Requirement 4, where we have addressed the Data Availability Statement in accordance with PLOS ONE’s requirements.

**Comment 3: Title is unclear due to two times PASC word appears.**

🡪 We thank the reviewer for this helpful comment. As described in our response to the Editor’s Minor Comment 4, we have revised the title to avoid redundancy and improve clarity.

**Reviewer #2 comment**

**Comment 1: First, the authors should clarify how they defined Long COVID. In this study, a participant was considered to have Long COVID if the PASC index was ≥12 at least once during follow-up. This does not mean that the score stayed high at every visit. This should be made clearer, especially when discussing persistent symptoms.**

🡪 Thank you for your thoughtful comment. We agree that the original wording may have implied that participants classified as having Long COVID had a PASC index ≥12 at every follow-up visit. Because defining Long COVID remains challenging, our study applied the RECOVER PASC index as a standardized symptom-based research definition. In this study, participants were classified into the Long COVID group if they had a PASC index ≥12 at least once during follow-up. Therefore, this classification indicates that the participant met the PASC index threshold at least once, but it does not imply that the participant continuously met the threshold at every assessment. We have revised the Methods, Results, and Discussion sections to clarify this definition.

At the same time, we acknowledge the limitations of this approach: because classification was based on ever meeting the threshold, the long COVID group may include participants whose scores exceeded the threshold only transiently. We have added these points to the Limitations section.

1) Revised) Methods, Line number 89–91

Accordingly, this classification indicates that participants ever met the PASC index threshold during follow-up and does not imply that the threshold was met at every assessment.

2) Revised) Discussion, Line number 228–231

This suggests that, once participants developed a sufficiently complex and multifaceted symptom burden to meet the PASC index threshold, their overall symptom burden tended to persist, even though their threshold status could fluctuate across visits, particularly when scores were close to the cutoff.

3) Revised) Discussion, Line number 282–286

Fourth, because Long COVID was defined by meeting the PASC index threshold at least once during follow-up, some participants may have exceeded the threshold only transiently. Thus, symptom persistence should be interpreted as persistence of a multifaceted symptom burden rather than sustained threshold-level scores at every assessment.

**Comment 2: the response rates were low and different at each time point, especially early in follow-up. This may affect the results. The authors should explain how missing data were handled and discuss this limitation more clearly.**

We thank the reviewer for this important comment. This issue substantially overlaps with the Editor’s Major Comment 1, and we have revised the manuscript accordingly.

We clarified in the Methods that missing PASC index values were not imputed. For the longitudinal GEE analysis, all available observations were included. For pairwise within-person comparisons of the continuous PASC index, analyses were restricted to participants with non-missing values at both time points being compared. We also added attrition analyses comparing baseline characteristics between responders and non-responders at each follow-up time point, and performed a complete-case sensitivity analysis restricted to participants who completed all four follow-up assessments.

The attrition analyses showed differential response patterns across follow-up time points, indicating that missingness was unlikely to be completely random. Therefore, we revised the Discussion to emphasize that changes in respondent composition may have influenced the observed prevalence trajectory and that temporal changes in the proportion of participants meeting the PASC index threshold should be interpreted cautiously. However, in the complete-case sensitivity analysis, pairwise within-person comparisons showed no significant change in the PASC index over time, supporting limited overall within-person change among participants with complete longitudinal follow-up. We also acknowledge that this sensitivity analysis does not fully eliminate the possibility of attrition bias.

1) Revised) Methods, Line number 106–110

Missing PASC index values were not imputed. For the longitudinal GEE analysis of the binary PASC index threshold, all available observations were included. For pairwise within-person comparisons of the continuous PASC index, analyses were restricted to participants with non-missing values at both time points being compared.

**Comment 3: there seems to be a difference between the abstract and the Results section. The abstract says that the PASC index significantly decreased in the non-Long COVID group, but the Results section reports no significant pairwise differences. This should be clarified.**

🡪 We thank the reviewer for pointing out this inconsistency. We re-examined the pairwise comparisons of the continuous PASC index and identified that the timepoint-specific p-values shown in Figure 2 had been computed using cross-sectional, unpaired comparisons. We have recomputed all pairwise comparisons using paired Wilcoxon signed-rank tests with Bonferroni correction, consistent with the Methods.

After this correction, the previously significant 1- versus 12-month comparison in the non-Long COVID group was no longer statistically significant. We have therefore revised the Abstract, Results, and Figure 2 to consistently state that pairwise within-person comparisons of the PASC index did not show significant changes over time.

1) Revised) abstracts, Line number 37–41

Participants classified as having Long COVID had consistently higher PASC index values across follow-up, while pairwise within-person comparisons showed no significant temporal changes in either the Long COVID or non-Long COVID group. These findings suggest that the overall symptom burden remained relatively stable over time, despite fluctuations in threshold status.

2) Revised) Results, Line number 162–171

In the long COVID group, the median PASC index was 14 (interquartile range, IQR, 9.25–18.8) at 1 month, 11 (7–16) at 3 months, 13.5 (8–18) at 6 months, and 14 (8–18) at 12 months. Pairwise within-person comparisons using paired Wilcoxon signed-rank tests showed no significant differences between any time points (1 vs. 3 months, p = 0.11; 3 vs. 6 months, p = 0.31; 6 vs. 12 months, p = 0.94; 1 vs. 12 months, p = 0.53), and none remained significant after Bonferroni correction. In the non-long COVID group, the median PASC index was 4 (3–7) at 1 month, 3 (1–6) at 3 months, 3 (0–6.25) at 6 months, and 2 (0–4) at 12 months, and pairwise comparisons likewise showed no significant differences (1 vs. 3 months, p = 0.60; 3 vs. 6 months, p = 0.73; 6 vs. 12 months, p = 0.36; 1 vs. 12 months, p = 0.54).

3) Revised) Fig 2

**Comment 4: Fourth, the Long COVID group was different from the non-Long COVID group at baseline. They were older and had more comorbidities, smoking, hospitalization, and COVID-19 treatment. These factors may affect symptoms and should be considered in the interpretation.**

Thank you for valuable comment. We agree that baseline differences between the Long COVID and non-Long COVID groups may have influenced symptom burden and should be considered when interpreting the findings.

As shown in Table 1, participants in the Long COVID group had a higher burden of baseline risk factors, including older age, smoking, comorbidities, hospitalization, and COVID-19-related treatment. These factors may contribute to persistent symptoms independently of Long COVID and may therefore affect both the PASC index and individual symptom frequencies. We have revised the Discussion to emphasize that the observed symptom burden should not be interpreted as being entirely attributable to SARS-CoV-2 infection or Long COVID itself.

At the same time, the attrition analyses added in response to the Editor’s Major Comment 1 showed differential response patterns over time. In particular, participants with greater comorbidity burden and more severe acute COVID-19-related features were less likely to contribute data at later follow-up assessments. This pattern may have attenuated, rather than exaggerated, the observed symptom burden at later time points. Therefore, although baseline differences remain an important limitation, the persistence of PASC index scores and symptoms over time is unlikely to be explained solely by baseline imbalance between the Long COVID and non-Long COVID groups. We have added this interpretation to the Discussion.

1) Revised) Discussion, Line number 286–294

Lastly, participants in the Long COVID group had a higher burden of baseline risk factors, including older age, smoking, comorbidities, hospitalization, and COVID-19-related treatment, which may have contributed to persistent symptoms independently of Long COVID itself. Conversely, attrition analyses suggested that more comorbid and severely affected participants were less likely to contribute data at later assessments, potentially attenuating the observed symptom burden over time. These opposing sources of bias complicate interpretation; baseline imbalance may have contributed to the observed differences but may not fully explain the persistence of symptom burden during follow-up.

**Comment 5: Fifth, this was a single-center study with many medically complex patients. The authors should be careful when discussing how generalizable the prevalence results are.**

We thank the reviewer for this important comment. We agree that the single-center design and the medically complex nature of our cohort limit the generalizability of the prevalence estimates.

Our study was conducted at a tertiary referral center and included a relatively high proportion of participants with comorbidities, immunocompromising conditions, hospitalization, and COVID-19-related treatment. Therefore, the observed prevalence of Long COVID based on the PASC index should not be interpreted as a population-level estimate or directly extrapolated to the general population of SARS-CoV-2 survivors.

We have revised the Discussion to emphasize that our findings describe the longitudinal kinetics of the RECOVER PASC index in a high-risk clinical cohort, rather than providing a generalizable estimate of Long COVID prevalence. This point is also related to the baseline differences between the Long COVID and non-Long COVID groups and the differential attrition patterns discussed in response to the Editor’s Major Comment 1 and Reviewer Comment 4. Although these factors limit the interpretation of prevalence, the complete-case sensitivity analysis and within-person comparisons support limited overall change in PASC index scores among participants with complete longitudinal follow-up. We have added this limitation and tempered the interpretation of the prevalence results accordingly.

1) Revised) Discussion, Line number 268–272

First, the relatively small, single-center tertiary-care cohort may have introduced selection bias and limited generalizability. Because our cohort included many medically complex patients, the observed prevalence of Long COVID should not be interpreted as a population-level estimate. Rather, our findings describe the longitudinal kinetics of the PASC index in a high-risk clinical cohort.

**Comment 6: Finally, the Data Availability Statement should be improved to meet PLOS ONE requirements.**

🡪 We thank the reviewer for this comment. Please refer to our response to Journal Requirement 4, where we have addressed the Data Availability Statement in accordance with PLOS ONE’s requirements.
