# Supplementary material for: Kinetics of the PASC Index in Long COVID
Source: PLoS One. 2026 Sep 8;21(9):e0357670. doi: 10.1371/journal.pone.0357670 (PMC13552729; doi:10.1371/journal.pone.0357670)
Supplement: Peer review history

# Peer review: Decision Letter 0

**DOI:** 10.1371/journal.pone.0357670.r001

**Authors:** Kokouvi Kassegne

23 Jun 2026

Dear Dr. Sung-Han Kim,

Thank you for submitting your manuscript to PLOS ONE. After careful consideration, we feel that it has merit but does not fully meet PLOS ONE’s publication criteria as it currently stands. Therefore, we invite you to submit a revised version of the manuscript that addresses the points raised during the review process.

Please submit your revised manuscript by  Aug 01 2026 11:59PM. If you will need more time than this to complete your revisions, please reply to this message or contact the journal office at plosone@plos.org. When you're ready to submit your revision, log on to https://www.editorialmanager.com/pone/ and select the 'Submissions Needing Revision' folder to locate your manuscript file.

- A letter that responds to each point raised by the academic editor and reviewer(s). You should upload this letter as a separate file labeled 'Response to Reviewers'.
- A marked-up copy of your manuscript that highlights changes made to the original version. You should upload this as a separate file labeled 'Revised Manuscript with Track Changes'.
- An unmarked version of your revised paper without tracked changes. You should upload this as a separate file labeled 'Manuscript'.

If you would like to make changes to your financial disclosure, please include your updated statement in your cover letter. Guidelines for resubmitting your figure files are available below the reviewer comments at the end of this letter.

If applicable, we recommend that you deposit your laboratory protocols in protocols.io to enhance the reproducibility of your results. Protocols.io assigns your protocol its own identifier (DOI) so that it can be cited independently in the future. For instructions see: https://journals.plos.org/plosone/s/submission-guidelines#loc-laboratory-protocols. Additionally, PLOS ONE offers an option for publishing peer-reviewed Lab Protocol articles, which describe protocols hosted on protocols.io. Read more information on sharing protocols at https://plos.org/protocols?utm_medium=editorial-email&utm_source=authorletters&utm_campaign=protocols.

As the corresponding author, your ORCID iD is verified in the submission system and will appear in the published article. PLOS supports the use of ORCID, and we encourage all coauthors to register for an ORCID iD and use it as well. Please encourage your coauthors to verify their ORCID iD within the submission system before final acceptance, as unverified ORCID iDs will not appear in the published article. *Only*  the individual author can complete the verification step; PLOS staff *cannot*  verify ORCID iDs on behalf of authors.

We look forward to receiving your revised manuscript.

Kind regards,

Kokouvi Kassegne

Academic Editor

PLOS One

Journal Requirements:

When submitting your revision, we need you to address these additional requirements.

1. Please ensure that your manuscript meets PLOS ONE's style requirements, including those for file naming. The PLOS ONE style templates can be found at

https://journals.plos.org/plosone/s/file?id=wjVg/PLOSOne_formatting_sample_main_body.pdf and

https://journals.plos.org/plosone/s/file?id=ba62/PLOSOne_formatting_sample_title_authors_affiliations.pdf

2. Thank you for stating in your Funding Statement:

“This work was supported by part of the Korea Health Technology Research and Development Project through the Korea Health Industry Development Institute (KHIDI), which is funded by the National Institute of Infectious Diseases, National Institute of Health, Republic of Korea (grant No. HD22C2045).?”

Please provide an amended statement that declares *all* the funding or sources of support (whether external or internal to your organization) received during this study, as detailed online in our guide for authors at http://journals.plos.org/plosone/s/submit-now. Please also include the statement “There was no additional external funding received for this study.” in your updated Funding Statement.

Please include your amended Funding Statement within your cover letter. We will change the online submission form on your behalf.

3. Thank you for stating the following financial disclosure:

“This work was supported by part of the Korea Health Technology Research and Development Project through the Korea Health Industry Development Institute (KHIDI), which is funded by the National Institute of Infectious Diseases, National Institute of Health, Republic of Korea (grant No. HD22C2045).”

Please state what role the funders took in the study. If the funders had no role, please state: "The funders had no role in study design, data collection and analysis, decision to publish, or preparation of the manuscript."

If this statement is not correct you must amend it as needed.

Please include this amended Role of Funder statement in your cover letter; we will change the online submission form on your behalf.

4. In the online submission form, you indicated that [Any inquiries regarding supporting data availability of this study should be directed to the corresponding author.].

All PLOS journals now require all data underlying the findings described in their manuscript to be freely available to other researchers, either 1. In a public repository, 2. Within the manuscript itself, or 3. Uploaded as supplementary information.

This policy applies to all data except where public deposition would breach compliance with the protocol approved by your research ethics board. If your data cannot be made publicly available for ethical or legal reasons (e.g., public availability would compromise patient privacy), please explain your reasons on resubmission and your exemption request will be escalated for approval.

5. If the reviewer comments include a recommendation to cite specific previously published works, please review and evaluate these publications to determine whether they are relevant and should be cited. There is no requirement to cite these works unless the editor has indicated otherwise.

**Additional Editor Comments** :

This prospective cohort study applies the NIH RECOVER PASC index to track long COVID symptom kinetics over 12 months. The finding that symptom burden persists in PASC patients while prevalence modestly declines is clinically relevant. However, several methodological concerns limit current interpretability.

Please provide a point-to-point response to the reviewers' comments and revise the manuscript according to show where revisions are made.

On a related note, please address the following issues:

**Major comments**

1. Response rate bias threatens validity. Only 34% completed the 1-month assessment versus 84% at 12 months. This inverse pattern is unusual and suggests selective non-response (e.g., asymptomatic individuals dropping out early). The authors must provide attrition analyses comparing baseline characteristics of responders vs. non-responders at each time point.

2. The "non-PASC" group contains probable PASC cases. Figure 3B shows fatigue, brain fog, and thirst are common in the <12 group. Using "never met criteria" as the comparator dilutes effect sizes. Consider sensitivity analyses defining non-PASC as consistently ≤11 across all time points.

3. Statistical reporting inconsistency. Abstract states prevalence decline from 27% to 18% was non-significant (p=0.16), but Results (line 136) reports a significant OR reduction at 3 months (p=0.04). Clarify which analysis is primary—GEE or pairwise comparisons?

**Minor comments**

4. Title redundancy. "Kinetics of PASC Index in Patients with PASC": suggest "Kinetics of the PASC Index in Long COVID"

5. Missing effect sizes. Provide Cohen's d or rank-biserial correlation for pairwise comparisons (not just p-values, which are underpowered given sample).

6. Figure 2B label error. "PASC index ≤12" is all individuals below threshold; this describes the non-PASC group, not the caption's claim of "consistently ≤12 across all time points."

7. Data availability statement insufficient. "Direct inquiries to corresponding author" does not meet PLOS ONE's requirement for full data access. Please deposit anonymized data in a public repository (e.g., Figshare, Dryad) with a DOI.

[Note: HTML markup is below. Please do not edit.]

Reviewers' comments:

Reviewer's Responses to Questions

**Comments to the Author**

1. Is the manuscript technically sound, and do the data support the conclusions?

Reviewer #1: Yes

Reviewer #2: Yes

**********

2. Has the statistical analysis been performed appropriately and rigorously?

Reviewer #1: Yes

Reviewer #2: Yes

**********

3. Have the authors made all data underlying the findings in their manuscript fully available?

The PLOS Data policy

Reviewer #1: No

Reviewer #2: Yes

**********

4. Is the manuscript presented in an intelligible fashion and written in standard English?

Reviewer #1: Yes

Reviewer #2: Yes

**********

Reviewer #1: The manuscript is well written and reported Kinetics of Post-Acute Sequelae of SARS-CoV-2 Infection (PASC)

Comment 1: Use consistent terminology e.g. COVID or COVID-19.

Comment 2: Full data should be made available as supplementary material.

Comment 3: Title is unclear due to two times PASC word appears

Reviewer #2: The manuscript discusses an important and timely topic: Long COVID symptoms over time using the PASC index. I found the article interesting and clinically relevant. The prospective design, 12-month follow-up, and use of a standardized PASC index are strengths.

I support publication after revision, but I have several comments.

First, the authors should clarify how they defined Long COVID. In this study, a participant was considered to have Long COVID if the PASC index was ≥12 at least once during follow-up. This does not mean that the score stayed high at every visit. This should be made clearer, especially when discussing persistent symptoms.

Second, the response rates were low and different at each time point, especially early in follow-up. This may affect the results. The authors should explain how missing data were handled and discuss this limitation more clearly.

Third, there seems to be a difference between the abstract and the Results section. The abstract says that the PASC index significantly decreased in the non-Long COVID group, but the Results section reports no significant pairwise differences. This should be clarified.

Fourth, the Long COVID group was different from the non-Long COVID group at baseline. They were older and had more comorbidities, smoking, hospitalization, and COVID-19 treatment. These factors may affect symptoms and should be considered in the interpretation.

Fifth, this was a single-center study with many medically complex patients. The authors should be careful when discussing how generalizable the prevalence results are.

Finally, the Data Availability Statement should be improved to meet PLOS ONE requirements.

Overall, this is a useful and meaningful study, and I believe it can be published after these issues are addressed.

**********

what does this mean?). If published, this will include your full peer review and any attached files.

If you choose “no”, your identity will remain anonymous but your review may still be made public.

**Do you want your identity to be public for this peer review?**  For information about this choice, including consent withdrawal, please see our Privacy Policy

Reviewer #1: No

Reviewer #2: **Yes:**  Yuriy Bisyuk

**********

[NOTE: If reviewer comments were submitted as an attachment file, they will be attached to this email and accessible via the submission site. Please log into your account, locate the manuscript record, and check for the action link "View Attachments". If this link does not appear, there are no attachment files.]

To ensure your figures meet our technical requirements, please review our figure guidelines: https://journals.plos.org/plosone/s/figures

You may also use PLOS’s free figure tool, NAAS, to help you prepare publication quality figures: https://journals.plos.org/plosone/s/figures#loc-tools-for-figure-preparation.

NAAS will assess whether your figures meet our technical requirements by comparing each figure against our figure specifications.

# Author comment: Author response to Decision Letter 1

**DOI:** 10.1371/journal.pone.0357670.r002

4 Aug 2026

Thank you for the helpful comments on our manuscript. Below we provide our responses to these comments.

Journal requirements

1. Please ensure that your manuscript meets PLOS ONE's style requirements, including those for file naming.

Thank you for your comment. We have carefully reviewed the manuscript to ensure that it meets PLOS ONE’s style requirements, including the requirements for file naming.

2. Thank you for stating in your Funding Statement: “This work was supported by part of Korea Health Technology Research and Development Project through the Korea Health Industry Development Institute (KHIDI), which is funded by the National Institute of Infectious Diseases, National Institute of Health, Republic of Korea (grant No. HD22C2045).?” Please provide an amended statement that declares *all* the funding or sources of support (whether external or internal to your organization) received during this study, as detailed online in our guide for authors at http://journals.plos.org/plosone/s/submit-now. Please also include the statement “There was no additional external funding received for this study.” in your updated Funding Statement. Please include your amended Funding Statement within your cover letter. We will change the online submission form on your behalf.

Thank you for your comment. We have amended the Funding Statement to declare all funding and sources of support received during this study. The amended Funding Statement has been included in the cover letter, as requested.

Funding/Support

We corrected the grant information to reflect the funding source that supported this study. This research was supported by the National Institute of Health (NIH), Republic of Korea, research project (2026-ER1901-00). There was no additional external funding received for this study. No additional internal funding or sources of support were received for this study.

3. Thank you for stating the following financial disclosure: “This work was supported by part of the Korea Health Technology Research and Development Project through the Korea Health Industry Development Institute (KHIDI), which is funded by the National Institute of Infectious Diseases, National Institute of Health, Republic of Korea (grant No. HD22C2045).” Please state what role the funders took in the study. If the funders had no role, please state: "The funders had no role in study design, data collection and analysis, decision to publish, or preparation of the manuscript. "If this statement is not correct you must amend it as needed. Please include this amended Role of Funder statement in your cover letter; we will change the online submission form on your behalf.

Thank you for your comment. We have amended the Role of Funder statement and included it in the cover letter, as requested.

Role of the Funder

The funders had no role in study design, data collection and analysis, decision to publish, or preparation of the manuscript.

4. In the online submission form, you indicated that [Any inquiries regarding supporting data availability of this study should be directed to the corresponding author.]. All PLOS journals now require all data underlying the findings described in their manuscript to be freely available to other researchers, either 1. In a public repository, 2. Within the manuscript itself, or 3. Uploaded as supplementary information. This policy applies to all data except where public deposition would breach compliance with the protocol approved by your research ethics board. If your data cannot be made publicly available for ethical or legal reasons (e.g., public availability would compromise patient privacy), please explain your reasons on resubmission and your exemption request will be escalated for approval.

Thank you for your comment. We have amended the Data Availability Statement in accordance with PLOS ONE’s data availability policy. The data underlying the findings of this study are now provided as Supporting Information files.

Data availability statement

All relevant data underlying the findings of this study are within the manuscript and its Supporting Information files.

Editor comment

Major comment 1: Response rate bias threatens validity. Only 34% completed the 1-month assessment versus 84% at 12 months. This inverse pattern is unusual and suggests selective non-response (e.g., asymptomatic individuals dropping out early). The authors must provide attrition analyses comparing baseline characteristics of responders vs. non-responders at each time point.

We thank the Editor for this important comment. First of all, the inverse pattern reflects the first enrollment timing of the participants in the study. Of a total of 183 patients, the first questionnaire was performed as follows; 63 (34.4%) at 1 month, 38 (20.8%) at 3 months, 22 (12.0%) at 6 months, and 60 (32.8%) at 12 months. Despite of this, we agree that the low and varying response rates across follow-up time points may introduce attrition bias and affect the interpretation of longitudinal prevalence estimates. To address this concern, we performed attrition analyses comparing baseline characteristics between responders and non-responders at each follow-up time point. Responders were defined as participants with a non-missing PASC index score at the corresponding assessment.

The attrition analyses showed that response patterns differed according to baseline clinical characteristics. At the 1-month assessment, responders had a greater burden of comorbidities and more severe acute COVID-19 than non-responders, including higher Charlson comorbidity index scores and higher proportions of chronic kidney disease, solid tumor, solid organ transplantation, hospitalization for COVID-19, and remdesivir treatment. Repeated SARS-CoV-2 infections were also more common among 1-month responders. Conversely, at the 12-month assessment, non-responders were older, more frequently male, and had higher Charlson comorbidity index scores and higher proportions of diabetes mellitus, solid tumor, hospitalization for COVID-19, and remdesivir treatment.

These findings indicate that missingness was unlikely to be completely random. In addition, many participants who contributed data at 12 months had not responded at the 1-month assessment, suggesting that 12-month respondents differed in composition from those assessed earlier. Therefore, the observed decline in the proportion of participants meeting the PASC index threshold may partly reflect differential non-response and changes in respondent composition, rather than natural symptom resolution alone.

To further examine whether this pattern was driven solely by changes in respondent composition, we performed a complete-case sensitivity analysis restricted to participants who completed all four follow-up assessments. Pairwise within-person comparisons using the Wilcoxon signed-rank test with Bonferroni correction showed no significant differences between 1 and 3 months, 3 and 6 months, 6 and 12 months, or 1 and 12 months (all adjusted p > 0.999), suggesting that symptom burden, as measured by the PASC index, remained persistent over 12 months among participants with complete longitudinal follow-up (S1 Fig).

We have added the attrition analyses as S4–7 Table and the complete-case sensitivity analysis to the Results section. We also revised the Limitations section to explicitly acknowledge this potential bias and its effect on prevalence estimates. Accordingly, we have toned down the interpretation of prevalence kinetics and clarified that our findings describe observed symptom trajectories among respondents in this cohort rather than unbiased population-level prevalence estimates.

1) Revised) Methods, Line number 79–83

A total of 183 participants were enrolled. The distribution of participants according to the timing of their first completed questionnaire was as follows: 63 (34.4%) at 1 month, 38 (20.8%) at 3 months, 22 (12.0%) at 6 months, and 60 (32.8%) at 12 months after infection. The overall numbers of participants who completed the questionnaire at each follow-up assessment were 63 of 183 (34.4%) at 1 month, 90 of 183 (49.2%) at 3 months, 106 of 183 (57.9%) at 6 months, and 154 of 183 (84.2%) at 12 months.

2) Revised) Methods, Line number 110–115

To assess potential attrition bias, we compared baseline characteristics between responders and non-responders at each follow-up time point. Responders were defined as participants with a non-missing PASC index score at the corresponding assessment. We also performed a complete-case sensitivity analysis restricted to participants who completed all four follow-up assessments, applying the same paired Wilcoxon signed-rank testing framework.

3) Revised) Results, Line number 180–190

Attrition and complete-case sensitivity analyses

Attrition analyses showed differential response patterns across follow-up time points (S4–7 Tables). Early responders tended to have greater comorbidity burden and more severe acute COVID-19-related features, whereas 12-month non-responders were older and had greater comorbidity and severity profiles than 12-month responders.

In the complete-case sensitivity analysis restricted to participants who completed all four assessments (n = 39), pairwise within-person comparisons using the Wilcoxon signed-rank test showed no significant differences in the PASC index across adjacent time points or between 1 and 12 months. The raw p-values were 0.31 for 1 vs. 3 months, 0.97 for 3 vs. 6 months, 0.78 for 6 vs. 12 months, and 0.50 for 1 vs. 12 months (S1 Fig). After Bonferroni correction for multiple comparisons, all adjusted p-values were >0.999.

4) Revised) Table S4–7. S1 Fig.

5) Revised) Discussion, Line number 272–280

Third, response rates varied across follow-up, particularly in the early phase, resulting in incomplete data and potential differential non-response. Attrition analyses suggested differential non-response, and changes in respondent composition may have influenced the observed prevalence trajectory. Therefore, temporal changes in the proportion of participants meeting the PASC index threshold should be interpreted cautiously. However, a complete-case sensitivity analysis showed no significant within-person change in the PASC index from 1 to 12 months, supporting limited overall change among participants with complete follow-up, although attrition bias cannot be excluded.

Major comment 2: The "non-PASC" group contains probable PASC cases. Figure 3B shows fatigue, brain fog, and thirst are common in the <12 group. Using "never met criteria" as the comparator dilutes effect sizes. Consider sensitivity analyses defining non-PASC as consistently ≤11 across all time points.

We thank the Editor for this important comment. We agree that participants with a PASC index below 12 may still have clinically relevant symptoms and should not be interpreted as completely asymptomatic controls. Symptoms such as fatigue, brain fog, and thirst are relatively non-specific and may occur without the total weighted PASC index reaching the diagnostic threshold. Therefore, the below-threshold group may include participants with subthreshold or probable PASC manifestations, which could dilute between-group differences.

To address this issue, we performed a complete-case sensitivity analysis using a more restrictive comparator definition. In this analysis, the non-long COVID group was defined as participants who completed all four follow-up assessments and had a PASC index ≤11 at every time point. Key symptoms, including fatigue, brain fog, and thirst, remained more frequent in the Long COVID group than in this non-Long COVID group. However, these symptoms were not completely absent among non-Long COVID participants, supporting the Editor’s concern that participants below the threshold may still have subthreshold symptoms. Overall, the findings were consistent with the main analysis, suggesting that our results were not driven solely by comparator misclassification, while still acknowledging the limitations of using a threshold-based classification.

This issue is also consistent with the way the RECOVER PASC index has been framed. The original RECOVER PASC index was proposed as an initial symptom-based classification framework rather than a definitive boundary separating symptomatic from asymptomatic individuals. Individuals who do not meet the score threshold may still require consideration of additional clinical or symptom data. Similarly, the updated RECOVER-Adult Long COVID Research Index introduced a “possible Long COVID” category, further supporting the concept that individuals below the threshold may still have subthreshold or probable Long COVID-related symptoms.

We have added this sensitivity analysis to the Results and added corresponding descriptions to the Methods and Discussion.

1) Revised) Methods, Line number 113–118

Separately, to address the possibility that participants below the PASC index threshold may have included subthreshold or probable Long COVID cases, we performed an additional complete-case sensitivity analysis using a more restrictive comparator definition. In this analysis, the non-Long COVID group was defined as participants who completed all four follow-up assessments and had a PASC index ≤11 at every time point.

2) Revised) Results, Line number 195–200

In the complete-case sensitivity analysis using a more restrictive comparator definition, key symptoms, including fatigue, brain fog, and thirst, remained more frequent in the Long COVID group than in the non-Long COVID group (S2 Fig). However, these symptoms were not completely absent among non-Long COVID participants, suggesting that participants below the PASC index threshold may still have subthreshold symptoms.

3) Revised) Discussion, Line number 249–252

Accordingly, comparisons using participants below the PASC index threshold as the reference group may have underestimated between-group differences. This limitation is inherent to threshold-based symptom classification, as individuals below the cutoff may still have possible or subthreshold Long COVID-related symptoms.

Major Comment 3. Statistical reporting inconsistency. Abstract states prevalence decline from 27% to 18% was non-significant (p=0.16), but Results reports a significant OR reduction at 3 months (p=0.04). Clarify which analysis is primary—GEE or pairwise comparisons.

Thank you for the valuable comment. We agree that the original wording did not clearly distinguish the primary longitudinal contrast from additional time-specific comparisons. We have clarified the statistical reporting to distinguish the longitudinal GEE analysis of the binary PASC index threshold from the pairwise Wilcoxon signed-rank tests used for the continuous PASC index. Our primary contrast of interest for the prevalence of Long COVID was the change between 1 and 12 months, reflecting the 12-month trajectory of PASC classification.

1) Revised) Methods, Line number 97–103

The primary longitudinal analysis evaluated changes in the proportion of participants with a PASC index ≥12 over time using generalized estimating equations (GEE) with an autoregressive(1) working correlation structure. Time was modeled as a categorical variable, with the 1-month assessment as the reference. Within-person changes in the continuous PASC index were evaluated using pairwise Wilcoxon signed-rank tests with Bonferroni correction for multiple comparisons.

2) Revised) Results, Line number 150–155

The prevalence of Long COVID was 27.0% (17/63) at 1 month, 16.7% (15/90) at 3 months, 21.7% (23/106) at 6 months, and 17.5% (27/154) at 12 months (Fig 1). In the GEE analysis using 1 month as the reference, the difference at 12 months was not statistically significant (OR = 0.64, p = 0.16). Although the odds of meeting the PASC index threshold were significantly lower at 3 months (OR = 0.46, p = 0.04), this reduction was not sustained at 6 months (OR = 0.57, p = 0.13) or 12 months.

Minor comment4: "Kinetics of PASC Index in Patients with PASC": suggest "Kinetics of the PASC Index in Long COVID.”

We thank the Editor for this helpful suggestion. We agree that the original title was redundant and have revised the title accordingly.

1) Revised) Title

Kinetics of the PASC Index in Long COVID

2) Revised) S

Attachment Submitted filename: Response letter_plos one.docx

# Peer review: Decision Letter 1

**DOI:** 10.1371/journal.pone.0357670.r003

**Authors:** Kokouvi Kassegne

20 Aug 2026

Kinetics of the PASC Index in Long COVID

PONE-D-25-23610R1

Dear Dr. Sung-Han Kim,

We’re pleased to inform you that your manuscript has been judged scientifically suitable for publication and will be formally accepted for publication once it meets all outstanding technical requirements.

Within one week, you’ll receive an e-mail detailing the required amendments. When these have been addressed, you’ll receive a formal acceptance letter and your manuscript will be scheduled for publication.

An invoice will be generated when your article is formally accepted. Please note, if your institution has a publishing partnership with PLOS and your article meets the relevant criteria, all or part of your publication costs will be covered. Please make sure your user information is up-to-date by logging into Editorial Manager at Editorial Manager® and clicking the ‘Update My Information' link at the top of the page. For questions related to billing, please contact billing support.

If your institution or institutions have a press office, please notify them about your upcoming paper to help maximize its impact. If they’ll be preparing press materials, please inform our press team as soon as possible -- no later than 48 hours after receiving the formal acceptance. Your manuscript will remain under strict press embargo until 2 pm Eastern Time on the date of publication. For more information, please contact onepress@plos.org.

Kind regards,

Kokouvi Kassegne

Academic Editor

PLOS One

Additional Editor Comments (optional):

The authors have revised the manuscript and responded to the reviewers' comment satisfactorily.

Reviewers' comments:

Reviewer's Responses to Questions

**Comments to the Author**

Reviewer #1: All comments have been addressed

Reviewer #2: All comments have been addressed

**********

2. Is the manuscript technically sound, and do the data support the conclusions?

Reviewer #1: Yes

Reviewer #2: Yes

**********

3. Has the statistical analysis been performed appropriately and rigorously?

Reviewer #1: Yes

Reviewer #2: Yes

**********

4. Have the authors made all data underlying the findings in their manuscript fully available?

The PLOS Data policy

Reviewer #1: Yes

Reviewer #2: Yes

**********

5. Is the manuscript presented in an intelligible fashion and written in standard English?

Reviewer #1: Yes

Reviewer #2: Yes

**********

Reviewer #1: Revisions are clear and reply is acceptable. The authors have made all changes in line of clarity and conciseness.

Reviewer #2: (No Response)

**********

what does this mean?). If published, this will include your full peer review and any attached files.

If you choose “no”, your identity will remain anonymous but your review may still be made public.

**Do you want your identity to be public for this peer review?**  For information about this choice, including consent withdrawal, please see our Privacy Policy

Reviewer #1: No

Reviewer #2: **Yes:**  Yuriy Bisyuk

**********

# Editor report: Acceptance letter

**DOI:** 10.1371/journal.pone.0357670.r004

**Authors:** Kokouvi Kassegne

PONE-D-25-23610R1

PLOS One

Dear Dr. Kim,

I'm pleased to inform you that your manuscript has been deemed suitable for publication in PLOS One. Congratulations! Your manuscript is now being handed over to our production team.

At this stage, our production department will prepare your paper for publication. This includes ensuring the following:

* All references, tables, and figures are properly cited

* All relevant supporting information is included in the manuscript submission,

* There are no issues that prevent the paper from being properly typeset

You will receive further instructions from the production team, including instructions on how to review your proof when it is ready. Please keep in mind that we are working through a large volume of accepted articles, so please give us a few days to review your paper and let you know the next and final steps.

Lastly, if your institution or institutions have a press office, please let them know about your upcoming paper now to help maximize its impact. If they'll be preparing press materials, please inform our press team within the next 48 hours. Your manuscript will remain under strict press embargo until 2 pm Eastern Time on the date of publication. For more information, please contact onepress@plos.org.

You will receive an invoice from PLOS for your publication fee after your manuscript has reached the completed accept phase. If you receive an email requesting payment before acceptance or for any other service, this may be a phishing scheme. Learn how to identify phishing emails and protect your accounts at https://explore.plos.org/phishing.

If we can help with anything else, please email us at customercare@plos.org.

Thank you for submitting your work to PLOS One and supporting open access.

Kind regards,

PLOS One Editorial Office Staff

on behalf of

Dr. Kokouvi Kassegne

Academic Editor

PLOS One
